# An Efficient Post-Quantum User Authentication Protocol for Wireless Sensor Networks Using NTRU

**DOI:** 10.3390/e28101049

**Published:** 2026-09-24

**Authors:** Wei Shen, Jiaqing Mo, Yuhua Lin

**Affiliations:** 1 School of Computer Science and Software, Zhaoqing University, Zhaoqing 526061, China; shenwei@zqu.edu.cn; 2 Center of Modern Educational Technology, Zhaoqing University, Zhaoqing 526061, China; lyh@zqu.edu.cn

**Keywords:** authentication, post-quantum, NTRU, authentication protocol, WSNs

## Abstract

Wireless sensor networks (WSNs) are pervasively employed in critical domains such as smart healthcare, industrial automation, and environmental surveillance, where secure access control is essential. User authentication protocols are vital for thwarting unauthorized intrusions, yet the vast majority of existing schemes rely on conventional public-key cryptography (such as ECC, RSA), which is provably insecure in the post-quantum era due to Shor’s algorithm. This motivates the design of post-quantum authentication that is also lightweight for resource-limited sensors. To address this challenge, we propose an efficient authentication and key agreement protocol based on Number Theory Research Unit (NTRU), a lattice-based cryptosystem offering proven resistance to quantum attacks. We have proven the correctness and security of our scheme through BAN logic and random oracle model and demonstrated through informal analysis that our scheme can resist various attack methods and possesses high security features. Moreover, we thoroughly evaluate our protocol against the most recent state-of-the-art schemes. The results indicate that our scheme has the lowest computational cost, and its communication cost is superior to that of most other schemes. Overall, our proposed protocol provides a robust, efficient, and future-proof authentication solution for WSNs, well-suited for deploying WSNs environments.

## 1. Introduction

Wireless sensor networks (WSNs) consist of numerous low-power resource-constrained sensor nodes deployed for environmental monitoring, industrial control, smart medical care and IoT systems [1]. The architecture of WSNs is shown in Figure 1. In WSNs, the sensors continuously collect sensitive data and transmit them to a user via a gateway node over wireless channels. However, the inherent openness of wireless channels makes user real-time access in WSNs vulnerable to various security and privacy threats [2]. User authentication acts as the core defense line to verify the legitimacy of communication entities. Without authentication mechanisms, attackers can forge sensor identities to inject false data or steal private monitoring information, which may lead to industrial system failures and leakage of patients’ private medical data [3]. Wireless sensor nodes within WSNs suffer from constrained energy supplies, limited computing capacity and narrow communication bandwidth, and they are generally deployed in unattended harsh field surroundings. These circumstances render traditional security mechanisms inapplicable to WSNs. In addition, the unprecedented progress of quantum computers has drastically undermined the security guarantees of traditional security frameworks, necessitating urgent countermeasures. Therefore, lightweight post-quantum secure authentication protocols are indispensable for the secure communication of WSNs.

### 1.1. Related Works

In WSNs environments, the sensitivity of transmitted data imposes stringent security requirements alongside severe resource limitations on sensor nodes. To tackle these conflicting challenges, numerous hash-function-based user authentication schemes have been developed in the literature [4,5,6,7,8,9,10,11]. Despite their computational efficiency, these protocols are not immune to adversarial threats—offline password guessing attacks in particular. Such vulnerabilities can either expose the established session key or cause it to fall short of the required security criteria, thereby undermining the assurance of secure key negotiation between the two communicating parties.

To overcome the inherent weaknesses of hash-based authentication schemes, Wang et al. advocated the adoption of public-key cryptographic operations as a more robust foundation for protocol design in general scenarios [12]. Among the available public-key alternatives, elliptic curve cryptography (ECC) stands out for its shorter key lengths and superior computational speed relative to RSA and other conventional systems, which has led to its extensive deployment in numerous authentication frameworks. In 2023, Sahoo et al. [13] proposed an improved three-factor wireless sensor authentication scheme based on ECC in a 5G environment to address the flaws present in previous schemes. However, we found that if the smartcard and biometric information are obtained by an attacker, the adversary can guess the user’s password, thus failing to provide three-factor security. Additionally, the scheme’s users use the same pseudo-identity UID for each login, allowing attackers to track user behavior, thus failing to provide anonymity. Meanwhile, Xie et al. [14] also pointed out that the Sahoo’s scheme was vulnerable to attacks, such as no data transmission security, transmitted data can be forged, user impersonation attack, and simultaneous impersonation attacks on gateway and sensor node, and proposed some improvement suggestions. In 2024, Saini et al. [15] proposed a three-factor security scheme to enhance the security of health data communication between doctors and patients in wireless healthcare sensor networks and believed their scheme to be efficient. However, since their scheme employs the computationally expensive ECC point multiplication for mutual authentication not only at the user and sensor ends but also at the gateway end, we believe their scheme is not as efficient as they claimed. To address issues such as impersonation attack, password guessing attacks, and internal attack in WSNs authentication protocol schemes, Huang [16] proposed a three-factor user authentication and key agreement scheme based on ECC. After analyzing this scheme, we believe that, similar to Sahoo et al.’s scheme, anonymity cannot be achieved because users use the same *TID_i_* for each login; similar to Saini et al.’s scheme, it is not efficient due to the extensive use of ECC point multiplication operations at the user end, gateway end, and sensor end. In 2025, Xu et al. [17] conducted a comprehensive cryptographic analysis of two authentication protocols [18,19] and demonstrated that they share four major vulnerabilities: offline password guessing attacks, node capture attacks, DoS service hijacking attacks, and the inability to achieve genuine multi-factor security and forward security. They also proposed an improvement scheme that employs a fuzzy verifier to compress the verification matching space to resist offline dictionary attacks and utilizes ECC to decouple the session key using public and private keys to circumvent node capture, thereby achieving forward security. In 2026, Yu et al. [20] proposed a lightweight authentication and key agreement protocol for resource-constrained IoT WSNs, utilizing ECC and PUF (physically unclonable function) to defend against common attacks and employing the Chinese Remainder Theorem to enable password recovery when users forget their passwords. However, we found that in their scheme, if the smartcard is obtained by the attacker, and the attacker also eavesdrops on the user login request messages on the public channel, by combining the secret parameters on the smartcard, the attacker can guess the user’s identity identifier and password, thereby being able to impersonate the user to log in to the system.

Due to its capability to produce seemingly random and unpredictable keys that effectively resist brute-force attacks, chaotic mapping is leveraged as a cryptographic primitive for building secure authentication schemes for WSNs in environments with stringent security requirements. Feng et al. [21] addressed the two core pain points in WSNs, namely the limited resources (computing, storage, and energy) of sensor nodes and the insecure public nature of communication channels. They proposed a lightweight two-factor authentication scheme based on Chebyshev chaotic mapping. However, this scheme claimed to be resistant to replay attacks and DoS attacks, but it did not use timestamps because it was actually impossible to achieve resistance to replay attack and DoS attack. According to our analysis, this scheme has the risk of desynchronization attack. In step 4 of the authentication stage, after the GW verifies the sensor successfully, it will update the temporary identity *PID_i_* in the database to the new alias *AID_i_*. At this time, if the last message is lost in the public channel (or intercepted and discarded by the attacker), then the user side still holds the old *PID_i_*, while the GW side has already replaced the old value with a new one. Then, when the user attempts to log in next time, he sends *PID_i_* to the GW, but the GW cannot match this identity in the database and will directly deny the service. Mo et al. [22] proposed a two-factor authentication protocol that combines the PUF and the Chebyshev chaotic mapping to address the overlooked physical cloning attack and node capture attacks in distributed wireless sensor networks. Wang et al. [23] proposed an authentication and key agreement protocol based on PUF, Chebyshev chaotic mapping, and three factors: biometric features, password, and smartcard, addressing the shortcomings of existing WSNs such as man-in-the-middle attack, replay attack, and physical cloning attacks.

With the development of quantum computing, cryptosystems capable of resisting quantum computer cracking are becoming increasingly important. Lattice-based cryptography is a new generation of cryptosystem designed to withstand this threat, and it is considered to be the most promising and fastest-standardizing anti-quantum cryptographic scheme at present. Currently, some authentication protocols have adopted lattice-based cryptosystems to achieve the authentication process and resist quantum attacks. Singh et al. [24] proposed a quantum-resistant authenticated key agreement protocol for WSNs. The core idea is to replace traditional discrete logarithm or large integer factorization problems with Ring Learning with Errors (RLWE) problems, aiming to resist the threat posed by future quantum computers to classical cryptosystems such as RSA and ECC. However, we have identified a significant logical error in their scheme, namely the misuse of the reconciliation function *ψ*_2_(,), which renders the scheme inoperable. In 2025, Jiang et al. [25] proposed a novel authentication protocol based on the NTRU cryptosystem to address two core challenges faced by underwater wireless sensor networks (UWSNs)—resource constraints (limited computation, storage, and energy of nodes) and the threat of quantum computing attacks. NTRU is a lattice-based lightweight public-key cryptosystem with security relying on the worst-case shortest vector problem (SVP) and closest vector problem (CVP) on lattices, offering fast encryption/decryption and low resource overhead [26]. Traditional authentication protocols, which either incur excessive computational overhead or fail to resist quantum attacks, struggle to balance security and efficiency in UWSNs. However, we find that the proposed scheme cannot resist an offline password guessing attack, and the use of NTRU encryption and decryption at the gateway side also makes the scheme computationally inefficient overall. Ahmad et al. [27] addressed the issue that existing post-quantum authentication schemes for the Internet of medical things (IoMT) environment, although quantum-resistant, incur excessive computational overhead and are thus unsuitable for resource-constrained IoMT devices. To this end, they proposed a lightweight quantum-resistant user authentication protocol based on the RLWE algorithm. However, upon analysis, we find that the scheme suffers from a fundamental flaw in mathematical and logical terms that makes it infeasible to execute. Specifically, in step 3 of the login and authentication phase, the sensor node does not possess *sid_i_* and *m*_i_, and consequently cannot recover *ν_i_*′ and *id_i_*′, thereby preventing the subsequent steps from being executed. Chaotic cryptography has also been explored for lightweight IoT sensing security [28,29]. Its attractive features include high sensitivity to initial conditions, pseudorandom keystreams, large key space, and resistance to statistical and differential attacks. However, inherent drawbacks remain: classical chaotic maps often have narrow chaotic parameter ranges, non-uniform dynamics, and periodic windows, which make keystreams predictable and vulnerable to known plaintext attacks. They also require abundant keystreams and complex key management, and they are symmetric primitives that cannot provide mutual authentication or key agreement under quantum attacks. Memristive-neuron models improve complexity and randomness but do not remove these limitations. In contrast, NTRU bases its security on hard lattice problems (SVP/CVP), offering quantum-resistant authentication and key agreement for WSNs.

### 1.2. Motivation and Contribution

From the previous analysis, it can be seen that currently a considerable number of authentication protocols are based on public key cryptography systems (such as ECC). A more fundamental concern is that the security of all traditional public-key primitives relies on the intractability of the integer factorization problem and the discrete logarithm problem, which have been proven by Shor to be easily broken by polynomial-time quantum algorithms [30]. Moreover, the current solution still has several security flaws, such as being unable to resist quantum attacks, offline password guessing attacks, and user impersonation attacks. Additionally, the computational cost and communication cost are relatively high, which imposes a significant burden on resource-constrained sensors. To address these issues, this paper proposes a lightweight security authentication protocol based on the NTRU algorithm for WSNs, which boasts low computation and communication costs, is capable of defending against common attacks, and is suitable for deploying on resource-constrained wireless sensors.

Our contributions are threefold:

(1) We propose an NTRU-based authentication scheme for WSNs, which not only resists quantum attack but also achieves low computation and communication costs.
(2) We have demonstrated that our scheme meets the security requirements through formal security proofs under BAN logic and random oracle model, as well as informal security analysis.
(3) We compare our scheme with the latest state-of-the-art related schemes in terms of computational and communication costs. The results show that our scheme is not only feasible but also efficient.

## 2. Preliminaries

### 2.1. Lattice

A lattice *L* is a discrete additive subgroup of *R^n^*. Given *m* linearly independent basis vectors *b*_1_, *b*_2_, …, *b_e_* ∈ *R^n^*, the lattice is formally defined as: *v* ∈ *L*, and

$$L\;=\left\{\sum_{i=1}^{m}z_{i}b_{i}|z_{i}\in Z\right\}$$

where *m* is the lattice rank, and *n* denotes the ambient dimension. All lattice points are integer linear combinations of basis vectors. Lattices possess a rich geometric structure, which gives rise to several computationally hard problems that form the foundation of post-quantum cryptography. Two of the most critical foundational problems are SVP and CVP—the Shortest Vector Problem and the Closest Vector Problem, respectively. SVP asks, given a lattice basis, to find a non-zero lattice vector *v* ∈ *L* that minimizes the Euclidean norm ||*v*||. CVP, given a basis of a lattice *L* and a target point *t* (not necessarily in the lattice), finds a lattice vector closest to *t*. Neither SVP nor CVP can be solved in polynomial time by classical or quantum algorithms, which distinguishes lattice cryptography from quantum-vulnerable ECC and chaotic maps cryptosystems for WSNs.

### 2.2. NTRU Cryptosystem

The lattice-based public-key cryptosystem NTRU was originally proposed by Hoffstein, Pipher, and Silverman back in 1998 [26]. Unlike RSA or ECC, whose security relies on integer factorization or discrete logarithms (vulnerable to Shor’s algorithm), NTRU’s security reduces to solving SVP (or CVP) in a convolutional polynomial ring. NTRU is renowned for its high efficiency and low resource consumption, making it ideal for WSNs.

The scheme operates in the ring *R* = *ℤ*[*x*]/(*x^n^* − 1), where n is a positive integer (typically a prime). Three parameters define an instantiation: *n*, a small modulus *p* (usually 2 or 3), and a large modulus *q* with gcd(*p,q*) = 1 and *q* ≫ *p*. Polynomial sets *L_f_*, *L_g_*, *Lᵣ*, and *L_m_* restrict coefficient ranges (e.g., {−1, 0, 1}) to ensure security and correctness.

Key Generation: Randomly choose *f* ∈ *L_f_* and *g* ∈ *L_g_* such that *f* is invertible modulo *p* and *q*. Compute *F_p_* ≡ *f*^−1^ (mod *p*) and *F_q_* ≡ *f*^−1^ (mod *q*). The public key is *h* ≡ *F_q_* · *g* (mod *q*), and the private key is (*f*, *F_q_*).

Encryption: For a plaintext *m* ∈ *L_m_*, select a random blinding polynomial *r* ∈ *Lᵣ* and compute

*e* = *p* · *r* · *h* + *m* (mod *q*)

Decryption: The recipient computes *a* = *f* · *e* (mod *q*), reduces its coefficients to (−*q*/2, *q*/2], and recovers

*m*′ = *F_p_* · *a* (mod *p*).

NTRU leverages fast cyclic polynomial multiplication to cut computation and memory overhead, perfectly matching the strict power and storage limits of low-power WSNs sensors, and serves as the post-quantum core of the proposed authentication protocol.

The NTRU ring-dimension n yields a critical security-performance trade-off. A larger n improves post-quantum resilience against SVP/CVP lattice attacks, yet raises polynomial-multiplication latency and memory consumption for constrained sensors. A smaller n accelerates NTRU encryption/decryption but reduces lattice security margins. This paper adopts a moderate-n setting balancing both metrics for WSN scenarios. Designers may adjust n for use-cases prioritizing either stronger security or lower latency.

## 3. Proposed Scheme

The scheme we propose includes system initialization phase, sensor registration phase, user registration phase, authentication phase, and password update phase. We will detail the specifics of each phase in this section, with the symbols and their meanings listed in Table 1.

### 3.1. System Initialization Phase

The system comprises three types of participants: *U_i_*, *GW*, *SN_j_*.

(1) *GW* publishes the public parameters {*p*, *q*, *n*, *n*_0_}, where {*p*, *q*, *n*, *n*_0_} is defined as required in Section 2.2, and *n*_0_ ∈ [24, 28] to all participating entities.
(2) *U_i_* selects polynomials *f_u_* ∈ *L_f_* and *g_u_* ∈ *L_g_* satisfying the condition of being invertible both modulo *q* and modulo *p*; that is, *f_u_* · $f_{{-1}_{u,p}}$ ≡ 1 (mod *p*), *f_u_* · $f_{{-1}_{u,q}}$ ≡ 1 (mod *q*), with the inverse element denoted by $f_{{-1}_{u,p}}$ and $f_{{-1}_{u,q}}$, respectively. *U_i_*’s public key is computed as *h_u_* = *p*·$f_{{-1}_{u,q}}$ · *g_u_* (mod *q*), and the corresponding private key is the pair (*f_u_*, $f_{{-1}_{u,p}}$).
(3) *SN_j_* selects polynomials *f_s_* ∈ *L_f_* and *g_s_* ∈ *L_g_* satisfying *f_s_* · $f_{{-1}_{s,p}}$ ≡ 1 (mod *p*), *f_s_* · $f_{{-1}_{s,q}}$≡ 1 (mod *q*). *SN_j_*’s public key is computed as *h_s_* = *p*·$f_{{-1}_{s,q}}$ · *g_s_* (mod *q*), and the corresponding private key is (*f_s_*, $f_{{-1}_{s,p}}$).

### 3.2. Sensor Registration Phase

The sensor *SN_j_* registers with the gateway GW before being deployed to the network. This phase establishes the necessary credentials for the sensor to participate in subsequent authentication operations.

(1) The sensor *SN_j_* chooses its identity *SID_j_* and transmits a registration request message {*SID_j_*, *h_s_*}to *GW* via a secure channel.
(2) Upon receiving the registration request, *GW* checks whether the identity *SID_j_* already exists in its database. If *SID_j_* is found, *GW* aborts the registration process. Otherwise, *GW* computes a secret key specifically for *SN_j_* as *K_j_* = *H*(*SID_j_*||*K_g_*), where *K_g_* is *GW*’s master secret key. *GW* stores the tuple {*SID_j_*, *K_j_*, *h_s_*} in its local database and transmits the message {*K_j_*, *h_s_*} to *SN_j_* through the secure channel.
(3) *SN_j_* stores {*K_j_*, *h_s_*} in its memory.

This phase is summarized in Figure 2.

### 3.3. User Registration Phase

The user *U_i_* registers with the gateway *GW* to obtain a personalized smartcard that will be used for subsequent authenticated access to sensors.

(1) *U_i_* keys their identity *U_IDi_* and password *U_PWi_*, selects a nonce *x* and computes *RID_i_* = *H*(*U_IDi_*||*x*), *F_i_* = *H*(*U_PWi_*||*x*) mod n_0_, and transmits a registration request message {*U_IDi_*, *RID_i_*} to *GW* via a secure channel.
(2) Upon receiving the registration request, *GW* verifies whether *U_IDi_* already exists in its user database. If the identity is already registered, the gateway aborts the registration. Otherwise, *GW* computes *K_i_* = *H*(*RID_i_*||*K_g_*) and stores {*U_IDi_ K_i_*, *h_u_*} in its database. Finally, *GW* sends a smartcard containing {*K_i_*, *h_s_*} to the user via a secure channel.

During registration over the secure channel, GW validates $h_{u}$ by checking that $deg\left(h_{u}\right)<n$, all coefficients lie in $Z_{q}$, $h_{u}\neq 0$, and $h_{u}$ conforms to the NTRU public-key format. Any malformed or maliciously constructed key causes GW to abort registration; thus, external adversaries cannot upload forged NTRU public keys. The function of deg() is to return the exponent of the highest non-zero degree term of a polynomial.

(3) Upon receiving the smartcard, *U_i_* computes *B_i_* = *H*(*U_IDi_*||*U_PWi_*) ⊕ *x*, $C_{i}=K_{i}$ ⊕ *F_i_* mod n_0_, deletes the *K_i_* from the smartcard, and then stores {*B_i_*, *C_i_*, *F_i_*} onto the smartcard.
  This phase is summarized in Figure 3.

### 3.4. Authentication Phase

This phase enables the user *U_i_* to establish mutual authentication with the sensor *SN_j_* through the mediation of the gateway *GW*, culminating in the establishment of a shared session key. The phase proceeds in five sequential steps.

(1) *U_i_* inserts the smartcard into a card reader and inputs *U_IDi_* and *U_PWi_*. The smartcard performs the following computations: *x* = *B_i_* ⊕ *H*(*U_IDi_*||*U_PWi_*), *F_i_’* = *H*(*U_PWi_*||*x*) mod *n*_0_ and then verifies whether *F_i_*′ equals the stored value *F_i_*. If the equality does not hold, the session is terminated immediately. Otherwise, the card computes *K_i_* = *C_i_* ⊕ *F_i_’* mod *n*_0_, selects a random nonce *a_i_* and generates a current timestamp *TS*_1_ and *r_u_* ∈ *Lᵣ*, computes *V*_1_ = *H*(*s_ug_*) ⊕ (*U_IDi_*||*SID_j_*||*TS*_1_), *V*_2_ = *p*·*h_s_*·*r_u_* + (*a_i_||U_IDi_*||*h_u_*), *m*_1_ = *H*(*U_IDi_*||*K_i_*||*h_u_*||*TS*_1_), and transmits the login request message {*V*_1_, *V*_2_, *m*_1_, *TS*_1_} to the *GW* over the public channel.
(2) Upon receiving the login request, *GW* checks the freshness of timestamp *TS*_1_ using the current time *TS*_1_*. If |*TS*_1_* − *TS*_1_| > *ΔT*, *GW* aborts the session; otherwise, *GW* extracts the identities by computing (*U_IDi_*||*SID_j_*||*TS*_1_) = *H*(*s_ug_*) ⊕ *V*_1_, retrieves the stored parameters {*K_i_*, *h_u_*} from the database using the extracted *U_IDi_*, and verifies whether *m*_1_ == *H*(*U_IDi_*||*K_i_*||*h_u_*||*TS*_1_). If the verification fails, *GW* terminates the session. Otherwise, *GW* retrieves *K_j_* from the database using the extracted *SID_j_* and computes *m*_2_ = H(*U_IDi_*||*K_j_*||*TS*_2_), where *TS*_2_ is the current timestamp. Finally, *GW* transmits the message {*V*_2_, *m*_2_, *TS*_2_} to *SN_j_* over the public channel.
(3) Upon receiving the message from the gateway, *SN_j_* checks the freshness of timestamp *TS*_2_ using the current time *TS*_2_*. If |*TS*_2_* − *TS*_2_| > *ΔT*, *SN_j_* terminates the session; otherwise, *SN_j_* decrypts *V*_2_ using the NTRU private key (*f_s_*, $f_{{-1}_{s,p}}$) to recover *a_i_||U_IDi_*||*h_u_* and verifies whether *m*_2_′ == *H*(*U_IDi_*||*K_j_*||*TS*_2_). If the verification fails, *SN_j_* aborts the session; otherwise, *SN_j_* selects a random nonce *b_j_* and generates a current timestamp *TS*_3_ and *r_s_* ∈ *Lᵣ*, computes *SK_u_*_−*s*_ = *H*(*a_i_*||*b_j_*||*U_IDi_*||*SID_j_*), *V*_4_ = *p* · *h_u_* · *r_s_* + *b_j_*, *m*_3_ = *H*(*K_j_*||*SID_j_*||*U_IDi_*||*TS*_3_), *m*_4_ = *H*(*b_j_*||*SID_j_*||*U_IDi_*||*TS*_3_), and transmits the message {*V*_4_, *m*_3_, *m*_4_, *TS*_3_} to *GW*.
(4) Upon receiving *SN_j_*’s response, *GW* checks the freshness of timestamp *TS*_3_ using the current time *TS*_3_*. If |*TS*_3_* − *TS*_3_| > *ΔT*, the gateway aborts the session; otherwise, *GW* verifies whether *m*_3_′ == *H*(*K_j_*||*SID_j_*||*U_IDi_*||*TS*_3_). If the verification fails, the gateway terminates the session; otherwise, *GW* picks up the current timestamp *TS*_4_, computes *m*_5_ == *H*(*K_i_*||*U_IDi_*||*TS*_4_), and transmits the message {*V*_4_, *m*_4_, *m*_5_, *TS*_4_} to *U_i_* over the public channel.
(5) Upon receiving the final message from *GW*, *U_i_* checks the freshness of timestamp *TS*_4_ using the current time *TS*_4_*. If |*TS*_4_* − *TS*_4_| > *ΔT*, the user aborts the session; otherwise, *U_i_* verifies whether *m*_5_′ == *H*(*K_i_*||*U_IDi_*||*TS*_4_). If the verification fails, the user terminates the session; otherwise, the user decrypts *V*_4_ to recover the random nonce *b_j_* and verifies whether *m*_4_′ == *H*(*b_j_*||*SID_j_*||*U_IDi_*||*TS*_3_). If the verification fails, the user terminates the session; otherwise, *U_i_* computes the session key *SK_u_*_−*s*_ = *H*(*a_i_*||*b_j_*||*U_IDi_*||*SID_j_*).

At the conclusion of this phase, both *U_i_* and *SN_j_* have mutually authenticated each other and established the shared session key $SK_{u-s}$, which can be used for subsequent secure communications.

This phase is summarized in Figure 4.

### 3.5. Password Update Phase

When the user wishes to change their password, the following steps are performed locally on the smartcard without requiring interaction with the gateway.

(1) *U_i_* inserts the smartcard and inputs *U_IDi_* and the current password *U_PWi_*. The smartcard computes *x* = *B_i_* ⊕ *H*(*U_IDi_*||*U_PWi_*), *F_i_*′ = *H*(*U_PWi_* ||*x*) mod *n*_0_, verifies whether *F_i_*′ equals the stored value $F_{i}$. If the verification fails, the session is terminated. Otherwise, the smartcard computes *K_i_* == *C_i_* ⊕ *F_i_’* mod *n*_0_.
(2) *U_i_* is prompted to input the new password $U_{new\;PWi}$. The smartcard performs the following computations: *F_i_^new^*= *H*($U_{new\;PWi}$||*x*) mod *n*, *B_i_^new^*= *H*(*U_IDi_*||$U_{new\;PWi}$) ⊕ *x*, *C_i_^new^ = K_i_*⊕*F_i_^new^*, replace the stored smartcard parameters (*B*_i_, *C*_i_, *F_i_*) with the new values (*B*_i_*^new^*, *C*_2_*^new^*, *F_i_^new^*).

The password update is now complete, and the user may use the new password *U _new PWi_* for subsequent login attempts.

## 4. Security Analysis

In this section, we assess the security of this scheme using BAN logic [31] and the random oracle model. Additionally, we provide heuristic analysis to demonstrate that this scheme can resist a variety of known attacks.

### 4.1. Formal Security Proof Under the BAN Logic

For brevity, we will not introduce the rules and symbols of BAN logic. Interested readers may refer to [32].

The actual authentication-phase messages are

$$\begin{array}{ll} M1 & {:U}_{i}\to GW:\{V_{1},V_{2},m_{1},{TS}_{1}\},\\ M2 & :GW\to SN_{j}:\{V_{2},m_{2},{TS}_{2}\},\\ M3 & :SN_{j}\to GW:\{V_{4},m_{3},m_{4},{TS}_{3}\},\\ M4 & :GW\to U_{i}:\{V_{4},m_{4},m_{5},{TS}_{4}\}. \end{array}$$

The relevant computations are

$$\begin{array}{ll} V_{1} & =H\left(s_{ug}\right)⊕\left(U_{IDi}∥SID_{j}∥{TS}_{1}\right),\\ V_{2} & ={ph}_{u}r_{u}+\left(a_{i}∥U_{IDi}∥h_{u}\right),\\ m_{1} & =H\left(U_{IDi}∥K_{i}∥h_{u}∥a_{i}∥{TS}_{1}\right),\\ m_{2} & =H\left(U_{IDi}∥K_{j}∥{TS}_{2}\right),\\ V_{4} & ={ph}_{u}r_{s}+b_{j},\\ m_{3} & =H\left(K_{j}∥SID_{j}∥U_{IDi}∥{TS}_{3}\right),\\ m_{4} & =H\left(b_{j}∥SID_{j}∥U_{IDi}∥{TS}_{3}\right),\\ m_{5} & =H\left(K_{i}∥U_{IDi}∥{TS}_{4}\right). \end{array}$$

In BAN logic, a hash/MAC authenticated by a shared key can be abstracted as a message encrypted/authenticated with that key. Thus, the idealized messages are

$$\begin{array}{l} M1\;(U_{i}\;\to \;\mathrm{GW}):\;\langle U_{IDi},SID_{j},a_{i},h_{u},{TS}_{1}\rangle_{K_{i}}\\ M2\;(\mathrm{GW}\;\to {\;\mathrm{SN}}_{j}):\langle U_{IDi},SID_{j},a_{i},h_{u},{TS}_{2}\rangle_{K_{j}}\\ M3\;({\mathrm{SN}}_{j}\;\to \;\mathrm{GW}):\;\langle SID_{j},U_{IDi},b_{j},{TS}_{3}\rangle_{K_{j}}\\ M4\;(\mathrm{GW}\;\to {\;U}_{i}):\langle SID_{j},b_{j},{TS}_{4}\rangle_{K_{i}} \end{array}$$

We formulate the following assumptions in accordance with the protocol’s initialization.

$$\begin{array}{l} (A1)\;\mathrm{GW}|\;\equiv {\;U}_{i}\overset{\;K_{i}}{↔}\mathrm{GW}\\ (A2)\;\mathrm{GW}|\;\equiv \;{\mathrm{SN}}_{j}\overset{\;K_{j}}{↔}\mathrm{GW}\\ (A3)\;U_{i}|\;\equiv \;U_{i}\overset{\;K_{i}}{↔}\mathrm{GW}\\ (A4)\;{\mathrm{SN}}_{j}|\;\equiv \;{\mathrm{SN}}_{j}\overset{\;K_{j}}{↔}\mathrm{GW}\\ (A5)\;U_{i}|\;\equiv \;\mathrm{GW}\;\Rightarrow \left(U_{i}\overset{\;\mathrm{SK}}{↔}{\mathrm{SN}}_{j}\right)\\ (A6)\;{\mathrm{SN}}_{j}|\;\equiv \;\mathrm{GW}\;\Rightarrow \left(U_{i}\overset{\;\mathrm{SK}}{↔}{\mathrm{SN}}_{j}\right)\\ (A7)\;U_{i}|\;\equiv \;{\mathrm{SN}}_{j}\;\Rightarrow \;b_{j}\\ (A8)\;{\mathrm{SN}}_{j}|\;\equiv \;U_{i}\;\Rightarrow \;a_{i}\\ (A9)\;\mathrm{GW}|\;\equiv \;\#\left({TS}_{1}\right)\\ (A10)\;{\mathrm{SN}}_{j}|\;\equiv \;\#\left({TS}_{2}\right)\\ (A11)\;\mathrm{GW}|\;\equiv \;\#\left({TS}_{3}\right)\\ (A12)\;U_{i}|\;\equiv \;\#\left({TS}_{4}\right)\\ (A13)\;\mathrm{GW}|\;\equiv \;\#\left(a_{i}\right)\\ (A14)\;U_{i}|\;\equiv \;\#\left(b_{j}\right)\\ (A15)\;{\mathrm{SN}}_{j}|\;\equiv \;U_{i}\overset{\;h_{u}}{↔}U_{i}\;\\ (A16)\;U_{i}|\;\equiv {\;\mathrm{SN}}_{j}\overset{\;h_{s}}{↔}{\mathrm{SN}}_{j}\\ (A17)\;{\mathrm{SN}}_{j}|\;\equiv \;\mathrm{priv}\left(h_{s}\right)\\ (A18)\;U_{i}|\;\equiv \;\mathrm{priv}\left(h_{u}\right)\\ (A19)\;GW|\;\equiv {\;U}_{i}\overset{\;h_{u}}{↔}U_{i}\\ (A20)\;GW|\;\equiv \;{\mathrm{SN}}_{j}\overset{\;h_{s}}{↔}{\mathrm{SN}}_{j}\\ (A21)\;\mathrm{GW}|\;\equiv \;\#U_{i}\overset{\;S_{\mathrm{ug}}}{↔}\mathrm{GW} \end{array}$$

The practical prerequisites for A1–A21 and their validity in real-world WSN deployments are listed in Table 2.

We define the goals of our scheme as follows:

$$\begin{array}{l} G1:U_{i}|\;\equiv \;(U_{i}\overset{\;SK}{↔}{SN}_{j})\\ G2:S_{j}|\;\equiv \;(U_{i}\overset{\;SK}{↔}{SN}_{j})\\ G3:U_{i}|\;\equiv \;{SN}_{j}|\;\equiv \;(U_{i}\;\overset{\;SK}{↔}{SN}_{j})\\ G4:{SN}_{j}|\;\equiv \;U_{i}|\;\equiv \;(U_{i}\;\overset{\;SK}{↔}{SN}_{j}) \end{array}$$

Based on the above definitions and BAN logic rules, we conduct the proof of the protocol’s validity as follows.

Step 1. From *M*1, GW receives $\mathrm{GW}⊲\;<{\;U}_{ID_{i}},\;{\mathrm{SID}}_{j},{\;a}_{i},\;h_{u},\;{TS}_{1}{\;>\;}_{K_{i}}.$

Step 2. By Message-Meaning Rule with assumption A1, we obtain

$$\mathrm{GW}|\equiv U_{i}|∼\;<U_{ID_{i}},\;{\mathrm{SID}}_{j},{\;a}_{i},{\;h}_{u},\;{TS}_{1}>.$$

Step 3. By Freshness Rule with A9, we obtain $\mathrm{GW}|\equiv \;\#\left(U_{ID_{i}},\;{\mathrm{SID}}_{j},{\;a}_{i},{\;h}_{u},\;{TS}_{1}\right).$

Step 4. By Nonce-Verification Rule from Steps 2 and 3, we obtain

$$\mathrm{GW}|\equiv U_{i}|\equiv \;<U_{ID_{i}},\;{\mathrm{SID}}_{j},{\;a}_{i},{\;h}_{u},\;{TS}_{1}>.$$

Step 5. By Belief Rule, we obtain $GW|\equiv U_{i}|\;\equiv \;a_{i},\mathrm{GW}|\;\equiv \;U_{i}|\;\equiv \;U_{ID_{i}},\mathrm{GW}|\;\equiv \;U_{i}|\;\equiv {\;h}_{u}.$

Step 6. From *M*2, ${\mathrm{SN}}_{j}$ receives ${\mathrm{SN}}_{j}⊲\;<{\;U}_{ID_{i}},\;a_{i},{\;h}_{u},\;{TS}_{2}{>}_{K_{j}}.$

Step 7. By Message-Meaning Rule with A4, we obtain

$${\mathrm{SN}}_{j}|\equiv \mathrm{GW}|∼<U_{ID_{i}},{{\mathrm{SID}}_{j},\;a}_{i},{\;h}_{u},\;{TS}_{2}\rangle .$$

Step 8. By Freshness Rule with A10, we obtain ${\mathrm{SN}}_{j}|\equiv \;\#\left(U_{ID_{i}},{\mathrm{SID}}_{j},\;a_{i},{\;h}_{u},\;{TS}_{2}\right).$

Step 9. By Nonce-Verification Rule, we obtain ${\mathrm{SN}}_{j}|\equiv \;\mathrm{GW}|\equiv \;<U_{ID_{i}},{{\mathrm{SID}}_{j},\;a}_{i},\;h_{u},\;{TS}_{2}>.$

Step 10. By Belief Rule, we obtain ${\mathrm{SN}}_{j}|\equiv \mathrm{GW}|\equiv a_{i}.$

Step 11. Since GW in Step 5 already believes that $U_{i}$ originated $a_{i}$, and GW is trusted by ${\mathrm{SN}}_{j}$ (A4), the sensor can infer that $a_{i}$ was indeed generated by $U_{i}$. Formally, combining Step 10 and the fact that GW believes $U_{i}$’s claim, we obtain ${\mathrm{SN}}_{j}|\equiv U_{i}|∼a_{i}.$

Step 12. By A8 and the Jurisdiction Rule, we obtain ${\mathrm{SN}}_{j}|\equiv a_{i}.$

Step 13. From M3, GW receives $\mathrm{GW}⊲\;<{\mathrm{SID}}_{j},\;U_{ID_{i}},\;b_{j},\;{TS}_{3}{>}_{K_{j}}.$

Step 14. By Message-Meaning Rule with A2, we obtain $\mathrm{GW}|\equiv {\mathrm{SN}}_{j}|∼<{\mathrm{SID}}_{j},\;U_{ID_{i}},{\;b}_{j},\;{TS}_{3}>.$

Step 15. By Freshness Rule with A13 and extending freshness to the whole message, we obtain

$$\mathrm{GW}|\equiv \;\#\left({\mathrm{SID}}_{j},{\;U}_{ID_{i}},{\;b}_{j},\;{TS}_{3}\right).$$

Step 16. By Nonce-Verification Rule, we obtain $\mathrm{GW}|\equiv {\mathrm{SN}}_{j}|\equiv \;<{\mathrm{SID}}_{j},{\;U}_{ID_{i}},\;b_{j},\;{TS}_{3}>.$

Step 17. By Belief Rule, we obtain $\mathrm{GW}|\equiv \;{\mathrm{SN}}_{j}|\equiv {\;b}_{j}.$

Step 18. From M4, $U_{i}$ receives $U_{i}⊲\;<{\mathrm{SID}}_{j},{\;b}_{j},\;{TS}_{4}{>}_{K_{i}}.$

Step 19. By Message-Meaning Rule with A3, we obtain $U_{i}|\equiv \mathrm{GW}|∼<{\mathrm{SID}}_{j},\;b_{j},\;{TS}_{4}>.$

Step 20. By Freshness Rule with A12, we obtain $U_{i}|\equiv \#\left({\mathrm{SID}}_{j},{\;b}_{j},\;{TS}_{4}\right).$

Step 21. By Nonce-Verification Rule, we obtain $U_{i}|\equiv \mathrm{GW}|\equiv \;<{\mathrm{SID}}_{j},\;b_{j},\;{TS}_{4}>.$

Step 22. By Belief Rule, we obtain $U_{i}|\equiv \mathrm{GW}|\equiv {\;b}_{j}.$

Step 23. From Step 17, GW believes that ${\mathrm{SN}}_{j}$ originated $b_{j}$. Since $U_{i}$ trusts GW’s judgment (A5), and the message is authenticated by GW, we can conclude that $U_{i}|\equiv {\mathrm{SN}}_{j}|∼b_{j}.$

Step 24. By A7 and the Jurisdiction Rule, we obtain $U_{i}|\equiv b_{j}.$

The session key is defined as $\mathrm{SK}\;=\;H\left(a_{i}\left|\right|b_{j}\left|\right|U_{ID_{i}}\left|\right|{\mathrm{SID}}_{j}\right).$

Step 25. From Step 12, ${\mathrm{SN}}_{j}|\equiv {\;a}_{i}$. The sensor also knows its own nonce $b_{j}$ and the identities $U_{ID_{i}},\;{\mathrm{SID}}_{j}.$ Therefore, ${\mathrm{SN}}_{j}|\equiv \left(a_{i},{\;b}_{j},\;U_{ID_{i}},\;{\mathrm{SID}}_{j}\right).$

Step 26. Since SK is a deterministic function of these values, the sensor believes that the key is shared with the user: ${\mathrm{SN}}_{j}|\equiv U_{i}\overset{\;\mathrm{SK}}{↔}{\mathrm{SN}}_{j}.$

Thus, G2 holds.

Step 27. From Step 24, $U_{i}|\equiv b_{j}.$ The user knows its own nonce $a_{i}$ and the identities. Hence,

$$U_{i}|\equiv \left(a_{i},\;b_{j},{\;U}_{ID_{i}},\;{\mathrm{SID}}_{j}\right).$$

Step 28. By the same reasoning, we obtain $U_{i}|\equiv U_{i}\overset{\;\mathrm{SK}}{↔}{\mathrm{SN}}_{j}.$

Thus, G1 holds.

Step 29. From Step 5, $\mathrm{GW}|\equiv U_{i}|\equiv a_{i}$; from Step 5, $\mathrm{GW}|\equiv {\mathrm{SN}}_{j}|\equiv b_{j}$; GW also knows ${\;U}_{ID_{i}}$ and ${\mathrm{SID}}_{j}$. Therefore,

$$\mathrm{GW}|\equiv U_{i}|\equiv \left(a_{i},{\;b}_{j},\;U_{ID_{i}},\;{\mathrm{SID}}_{j}\right)$$

$$\mathrm{GW}|\equiv {\mathrm{SN}}_{j}|\equiv \left(a_{i},{\;b}_{j},\;U_{ID_{i}},\;{\mathrm{SID}}_{j}\right)$$

Since SK is a deterministic function of these values,

$$\mathrm{GW}|\equiv U_{i}|\equiv \left(U_{i}\overset{\;\mathrm{SK}}{↔}{\mathrm{SN}}_{j}\right)$$

$$\mathrm{GW}|\equiv {\mathrm{SN}}_{j}|\equiv \left(U_{i}\overset{\;\mathrm{SK}}{↔}{\mathrm{SN}}_{j}\right)$$

Step 30. From Step 21, $U_{i}|\equiv \mathrm{GW}|\equiv <{\mathrm{SID}}_{j},\;b_{j},\;{TS}_{4}>$. By A5, $U_{i}$ trusts GW’s jurisdiction over the session key. Combined with Step 29, where GW believes that ${\mathrm{SN}}_{j}$ believes the session key, $U_{i}$ can conclude that

$$U_{i}|\equiv {\mathrm{SN}}_{j}|\equiv \left(U_{i}\overset{\;\mathrm{SK}}{↔}{\mathrm{SN}}_{j}\right)$$

Thus, G3 holds.

Step 31. From Step 9,${\;\mathrm{SN}}_{j}|\equiv \mathrm{GW}|\equiv \;<U_{ID_{i}},{{\mathrm{SID}}_{j},\;a}_{i},\;h_{u},\;{TS}_{2}>$. By A6, ${SN}_{j}$ trusts GW’s jurisdiction over the session key. Combined with Step 29, where GW believes that $U_{i}$ believes the session key, $SN_{j}$ can conclude

$${\mathrm{SN}}_{j}|\equiv U_{i}|\equiv \left(U_{i}\overset{\;\mathrm{SK}}{↔}{\mathrm{SN}}_{j}\right)$$

Thus, G4 holds.

Therefore, the achievement of Goals 1–4 implies that the proposal enables *U_i_* and *SN_j_* to mutually authenticate each other, while simultaneously negotiating a shared session key for subsequent communications.

### 4.2. Formal Security Proof Under Random Oracle Model

The security of the proposed scheme is formally validated in the random oracle model in this subsection. For conciseness, we adopt the adversary model of [33,34] without loss of generality.

**Theorem** **1.** *Let Π denote the proposal, and let the hash function *$H:\{0,1{\}}^{*}\to \{0,1{\}}^{l}$ *be modeled as a random oracle, where l is the bit-length of hash output. Let the NTRU encryption scheme be instantiated with public parameters* $\left(p,q,n,n_{0},L_{f},L_{g},L_{r}\right)$*, and assume that the NTRU decryption problem (NDP) is hard: for any probabilistic polynomial-time algorithm running in time t, its success probability in inverting an NTRU ciphertext is at most Adv_NDP_(t), which is a negligible function in the security parameter.*

*For any probabilistic polynomial-time adversary* A* against the authenticated key exchange (AKE) security of Π, making at most* $q_{s}$* Send queries, *$q_{e}$* Execute queries, and* $q_{h}$* hash queries to the random oracle, the advantage of* A* in winning the Test query (distinguishing the real session key from a random string of equal length) is bounded by*

$$\begin{array}{ll} {\mathrm{Adv}}_{\Pi }^{\mathrm{AKE}}\left(A\right) & \leq \frac{2q_{s}+q_{h}^{2}+{\left(q_{s}+q_{e}\right)}^{2}}{2^{l}}+\frac{{\left(q_{s}+q_{e}\right)}^{2}}{2\left(n_{0}-1\right)}\\ & \;+2q_{h}\cdot max\;\left\{\;\frac{q_{s}}{\left|D_{\mathrm{ID}}\right|},\;\frac{q_{s}}{\left|D_{\mathrm{PW}}\right|},\;\frac{1}{n_{0}}\right\}\\ & \;+2q_{h}\cdot {\mathrm{Adv}}_{\mathrm{NDP}}\left(t+\left(2q_{s}+6q_{e}\right)\;T_{\mathrm{ntru}}\right)\\ & \;+4q_{h}\;{\left(q_{s}+q_{e}\right)}^{2}\cdot {\mathrm{Adv}}_{\mathrm{NDP}}\;\left(t+\left(q_{s}+q_{e}\right)\;T_{\mathrm{ntru}}\right), \end{array}$$

*where* ${\mathrm{Adv}}_{\Pi }^{\mathrm{AKE}}\left(A\right)$ *denotes the advantage of* A *in breaking security of AKE in Π, n_0_ ∈ [24, 28] denotes the public modulus used in the fuzzy verifier,* $\left|D_{\mathrm{ID}}\right|$ *denotes the size of dictionary space of possible user identities,* ${|D}_{\mathrm{PW}}|$ *denotes the size of dictionary of possible user passwords,* $T_{\mathrm{ntru}}$ *denotes the time required for one NTRU encryption or decryption operation, and* $q_{s},q_{e},q_{h}$ *denote the number of Send, Execute, and hash queries, respectively.*

*The protocol is considered AKE-secure if this advantage is negligible in the security parameter* l* (and in the NTRU dimension* n*), under the stated hardness assumption.*

**Proof** **of** **Theorem** **1.** We prove the theorem by a sequence of games $G_{0},G_{1},\ldots ,G_{5}$ starting from the real execution and ending in an idealized game where the session key is completely independent of the adversary’s view. Let $S_{i}$ denote the event that A correctly guesses the hidden bit b in the Test query in game $G_{i}$. The advantage in game $G_{i}$ is ${\mathrm{Adv}}_{i}=2Pr\left[S_{i}\right]-1$, and we bound the differences $\left|Pr\left[S_{i}\right]-Pr\left[S_{i-1}\right]\right|$ for each transition.

Game $G_{0}$: This is the actual execution of Π under the random oracle model. All oracles—including the hash function, NTRU encryption/decryption, and timestamp checks—are implemented exactly as specified. Thus, we have

(1) $${\mathrm{Adv}}_{\Pi }^{\mathrm{AKE}}\left(A\right)=2Pr\left[S_{0}\right]-1$$

Game $G_{1}$: We replace the real hash function by a random oracle that maintains a list $L_{H}$ of all input–output pairs. Every new hash query is answered with a uniformly random string in $\{0,1{\}}^{l}$, and the answer is recorded for consistency. The NTRU operations are simulated using the actual public/private keys. This game is perfectly indistinguishable from $G_{0}$ from the adversary’s perspective; hence,

(2) $$Pr\left[S_{1}\right]=Pr\left[S_{0}\right]$$

Game $G_{2}$: We abort the game if any of the following collisions occur:

- **Hash collisions**: Two distinct inputs yield the same output. The probability of this event among $q_{h}$ hash queries is at most $q_{h}^{2}/2^{l+1}$.
- **Nonce collisions**: The random values $a_{i},b_{j},r_{u},r_{s}$ (drawn from spaces of size at least $n_{0}-1$) repeat across different sessions. For $\left(q_{s}+q_{e}\right)$ sessions, the union bound gives at most ${\left(q_{s}+q_{e}\right)}^{2}/\left(2\left(n_{0}-1\right)\right)$.
- **Transcript collisions**: Two different executions produce identical full messages, which could allow the adversary to replay or confuse sessions. This is bounded by the same term ${\left(q_{s}+q_{e}\right)}^{2}/2^{l+1}$.

Summing these probabilities gives

(3) $$\left|Pr\left[S_{2}\right]-Pr\left[S_{1}\right]\right|\leq \frac{q_{h}^{2}+{\left(q_{s}+q_{e}\right)}^{2}}{2^{l+1}}+\frac{{\left(q_{s}+q_{e}\right)}^{2}}{2\left(n_{0}-1\right)}$$

Game $G_{3}$: In this game, we rule out the possibility that A produces a valid authentication tag $m_{1},m_{2},m_{3},m_{4},$ or $m_{5}$ without having queried the corresponding hash input. Each tag involves a secret value (e.g., $K_{i}$, $K_{j}$, or the nonce $b_{j}$) that is unknown to the adversary. If A attempts to guess a tag without querying the oracle, the success probability per attempt is $1/2^{l}$. Across $q_{s}$ Send queries and $q_{h}$ hash queries, the total forgery probability is bounded by $q_{s}q_{h}/2^{l}\leq q_{h}/2^{l}$ (assuming $q_{s}\leq q_{h}$, which is typical). Thus,

(4) $$\left|Pr\left[S_{3}\right]-Pr\left[S_{2}\right]\right|\leq \frac{q_{h}}{2^{l}}$$

Game $G_{4}$: We now address the two main adversarial strategies that could compromise the session key: (i) recovering the ephemeral nonces $a_{i}$ and $b_{j}$ from the NTRU ciphertexts $V_{2}$ and $V_{4}$ and (ii) performing an offline dictionary attack against the user’s identity/password using the smartcard data. We analyze these two cases separately.

Case 1: Reduction to NDP

To compute the session key

$$SK=H\left(a_{i}∥b_{j}∥U_{IDi}∥SID_{j}\right),$$

the adversary must know both $a_{i}$ and $b_{j}$ (or at least the hash preimage, which is as hard as inverting H(). The only way to obtain them without guessing is to decrypt the NTRU ciphertexts:

- $V_{2}=h_{s}\cdot r_{u}+\left(a_{i}∥U_{IDi}∥h_{u}\right)$ requires the sensor’s private key $\left(f_{s},f_{s,p}^{-1}\right)$;
- $V_{4}=h_{u}\cdot r_{s}+b_{j}$ requires the user’s private key $\left(f_{u},f_{u,p}^{-1}\right)$.

We construct a reduction B that embeds a challenge NTRU ciphertext (say, targeting $h_{s}$) into the simulation. If A correctly guesses the session key, then, with probability at least $1/q_{h}$, it has queried the oracle on the correct $a_{i}$ (and $b_{j}$). By inspecting the hash list, B extracts the plaintext and solves the NDP instance. A symmetric reduction handles the case where $V_{4}$ is targeted. The total success probability of the reduction is bounded by the adversary’s advantage multiplied by $q_{h}$, which yields the term

$$q_{h}\cdot {\mathrm{Adv}}_{\mathrm{NDP}}\left(t+\left(2q_{s}+6q_{e}\right)\;T_{\mathrm{ntru}}\right).$$

Case 2: Offline Guessing with Fuzzy Verifier

Suppose A compromises the smartcard and obtains $\left\{B_{i},C_{i},F_{i}\right\}$. To verify a candidate identity/password pair $\left(U_{IDi}^{*},U_{PWi}^{*}\right)$, it computes

$$x^{*}=B_{i}⊕H\left(U_{IDi}^{*}∥U_{PWi}^{*}\right),\;F_{i}^{*}=H\left(U_{PWi}^{*}∥x^{*}\right)\;mod\;n_{0}.$$

The guess is accepted if $F_{i}^{*}=F_{i}$. Since the hash output is uniformly distributed, the probability that a wrong pair satisfies this equality is exactly $1/n_{0}$. Thus, for $q_{s}$ online guesses, the success probability of guessing the correct credentials is at most

$$max\left\{\frac{q_{s}}{\left|D_{\mathrm{ID}}\right|},\;\frac{q_{s}}{\left|D_{\mathrm{PW}}\right|},\;\frac{1}{n_{0}}\right\},$$

where the first two terms correspond to dictionary attacks when one factor (identity or password) is known, and the third is the false-acceptance rate of the fuzzy verifier. Since the adversary may attempt this for each hash query, we multiply by $q_{h}$.

Combining both cases gives

(5) $$\left|Pr\left[S_{4}\right]-Pr\left[S_{3}\right]\right|\leq q_{h}\cdot max\left\{\frac{q_{s}}{\left|D_{\mathrm{ID}}\right|},\frac{q_{s}}{\left|D_{\mathrm{PW}}\right|},\frac{1}{n_{0}}\right\}+q_{h}\cdot {\mathrm{Adv}}_{\mathrm{NDP}}\left(t+\left(2q_{s}+6q_{e}\right)T_{\mathrm{ntru}}\right)$$

Game $G_{5}$: In this final game, we consider the adversary’s ability to compromise session keys established before it obtains long-term secrets. Specifically, A may issue a Corrupt(GW) query and obtain the gateway’s long-term master secret $K_{g}$. From $K_{g}$, A can derive any gateway-side secret key of the form

$$K_{i}=H\left(RID_{i}∥K_{g}\right),\;\;K_{j}=H\left(SID_{j}∥K_{g}\right).$$

Thus, A can verify or forge all MACs/tags $m_{1},m_{2},m_{3},m_{4},m_{5}$ that are computed with $K_{i}$ or $K_{j}$. However, A does **not** obtain the NTRU private keys

$$\left(f_{u},f_{u}^{-1}\right)\;\mathrm{of}\;U_{i},\;\left(f_{s},f_{s}^{-1}\right)\;\mathrm{of}\;SN_{j},$$

nor the ephemeral nonces $a_{i},b_{j},r_{u},r_{s}$, which are assumed to be erased after the session. This is consistent with the standard forward-secrecy threat model: the long-term key of the gateway is leaked, but the per-node private keys and ephemeral session state remain protected.

We claim that even with $K_{g}$, A cannot compute the historical session key

$$SK=H\left(a_{i}∥b_{j}∥U_{IDi}∥SID_{j}\right).$$

The reason is that SK does not depend on $K_{g}$, $K_{i}$, or $K_{j}$. It depends only on the ephemeral nonces $a_{i}$ and $b_{j}$ and the public identities $UID_{i},SID_{j}$. In the transcript, these nonces are protected by NTRU encryption:

$$V_{2}=h_{s}r_{u}+\left(a_{i}∥U_{IDi}∥h_{u}\right),$$

$$V_{4}=h_{u}r_{s}+b_{j}.$$

Recovering $a_{i}$ from $V_{2}$ requires the sensor’s private key $\left(f_{s},f_{s}^{-1}\right)$. Recovering $b_{j}$ from $V_{4}$ requires the user’s private key $\left(f_{u},f_{u}^{-1}\right)$. Neither private key is derivable from $K_{g}$. Therefore, from A’s view, $a_{i}$ and $b_{j}$ are hidden by the NDP.

To distinguish the real SK from a random string of the same length, A must query the random oracle H on the correct tuple

$$\left(a_{i},b_{j},U_{IDi},SID_{j}\right).$$

Without such a query, the value of $H\left(\cdot \right)$ is uniformly random and independent of A’s view. We now bound the probability that A makes this query.

Let Q be the event that A queries H on the correct tuple for the Test session. We construct a reduction B that solves an NDP instance using A. B receives an NDP challenge $\left(h^{*},c^{*}\right)$. It guesses

- One session among the $q_{s}+q_{e}$ executed sessions;
- One hash query among the $q_{h}$ queries made by A.

B embeds $c^{*}$ as $V_{2}$ (if targeting $f_{s}$) or $V_{4}$ (if targeting $f_{u}$) in the guessed session. All other values are simulated using $K_{g}$ and the known protocol logic. If A queries H on the correct tuple for that session, then B reads $a_{i}$ (or $b_{j}$) from the query input and outputs it as the NDP plaintext.

The probability that B’s guess is correct is at least

$$\frac{1}{{\left(q_{s}+q_{e}\right)}^{2}}q_{h}.$$

Therefore,

$$Pr\left[Q\right]\leq {\left(q_{s}+q_{e}\right)}^{2}q_{h}\cdot {Adv}_{NDP}\left(t+\left(q_{s}+q_{e}\right)T_{ntru}\right).$$

If both $a_{i}$ and $b_{j}$ must be recovered, a factor of 2 may be included, yielding

$$Pr\left[Q\right]\leq 2{\left(q_{s}+q_{e}\right)}^{2}q_{h}\cdot {Adv}_{NDP}\left(t+\left(q_{s}+q_{e}\right)T_{ntru}\right).$$

In Game $G_{5}$, if Q does not occur, the session key is replaced by a uniformly random string. Hence,

(6) $$Pr\left[S_{5}\right]=\frac{1}{2}$$

Consequently,

(7) $$\left|Pr\left[S_{5}\right]-Pr\left[S_{4}\right]\right|\leq Pr\left[Q\right]\leq 2{\left(q_{s}+q_{e}\right)}^{2}q_{h}\cdot {Adv}_{NDP}\left(t+\left(q_{s}+q_{e}\right)T_{ntru}\right)$$

Combining (1)~(7), we have

$$\begin{array}{ll} {\mathrm{Adv}}_{\Pi }^{\mathrm{AKE}}\left(A\right) & =2Pr\left[S_{0}\right]-1\\ & \leq 2\sum_{i=1}^{5}\left|Pr\left[S_{i}\right]-Pr\left[S_{i-1}\right]\right|\\ & \leq \frac{2q_{s}+q_{h}^{2}+{\left(q_{s}+q_{e}\right)}^{2}}{2^{l}}+\frac{{\left(q_{s}+q_{e}\right)}^{2}}{2\left(n_{0}-1\right)}\\ & \;+2q_{h}\cdot max\left\{\;\frac{q_{s}}{\left|D_{\mathrm{ID}}\right|},\;\frac{q_{s}}{\left|D_{\mathrm{PW}}\right|},\;\frac{1}{n_{0}}\right\}\\ & \;+2q_{h}\cdot {\mathrm{Adv}}_{\mathrm{NDP}}\;\left(t+\left(2q_{s}+6q_{e}\right)\;T_{\mathrm{ntru}}\right)\\ & \;+4q_{h}\;{\left(q_{s}+q_{e}\right)}^{2}\cdot {\mathrm{Adv}}_{\mathrm{NDP}}\left(t+\left(q_{s}+q_{e}\right)\;T_{\mathrm{ntru}}\right) \end{array}$$

This completes the proof. □

### 4.3. Informal Security Analysis

This section provides an informal security evaluation, verifying the proposed scheme’s robustness against diverse attacks and its compliance with essential privacy and security requirements. In addition, we compared our scheme with four latest schemes in terms of security attributes, and the results are presented in Table 3.

#### 4.3.1. User Impersonation Attack

An adversary attempting to impersonate user $U_{i}$ must forge a valid login request $\left\{V_{1},V_{2},m_{1},{TS}_{1}\right\}$. This requires knowledge of

- The identity $U_{IDi}$ and password $U_{PWi}$ to recover $x=B_{i}⊕H\left(U_{IDi}∥U_{PWi}\right)$,
- The secret $K_{i}=C_{i}⊕H\left(U_{PWi}∥x\right)\;mod\;n_{0}$,
- The gateway’s secret $s_{ug}$ to construct $V_{1}=H\left(s_{ug}\right)⊕\left(U_{IDi}∥SID_{j}\right)$,
- The ephemeral nonce $a_{i}$ and the NTRU public key $h_{s}$ to build $V_{2}$.

Even if the adversary obtains the smartcard and extracts $\left\{B_{i},C_{i},F_{i}\right\}$, he cannot compute x without $U_{IDi}$ and $U_{PWi}$. The fuzzy verifier $F_{i}=H\left(U_{PWi}∥x\right)\;mod\;n_{0}$ ensures that a guessed password/identity pair cannot be verified offline with certainty, as the modulo operation yields approximately $\left|D_{ID}\right|\cdot \left|D_{PW}\right|/n_{0}$ false positives. To distinguish the correct pair, the adversary must launch an online login attempt, which is detectable and limited by timestamp checks. Without $K_{i}$, forging $m_{1}=H\left(U_{IDi}∥K_{i}∥h_{u}∥{TS}_{1}\right)$ is infeasible due to the one-way property of H(). Hence, the scheme resists user impersonation.

#### 4.3.2. GW Impersonation Attack

To impersonate the gateway GW, the adversary must produce valid messages $\left\{V_{2},m_{2},{TS}_{2}\right\}$ to $S_{j}$ and $\left\{V_{4},m_{4},m_{5},{TS}_{4}\right\}$ to $U_{i}$. These require

- $m_{2}=H\left(U_{IDi}∥K_{j}∥{TS}_{2}\right)$, where $K_{j}=H\left(SID_{j}∥K_{g}\right)$ is the sensor’s long-term key known only to GW and $S_{j}$,
- $m_{5}=H\left(K_{i}∥U_{IDi}∥{TS}_{4}\right)$, where $K_{i}=H\left(RID_{i}∥K_{g}\right)$ is known only to GW and the legitimate user,
- The NTRU ciphertexts $V_{2}$ and $V_{4}$, which are constructed using the sensor’s public key $h_{s}$ and the user’s public key $h_{u}$, respectively.

An adversary without the master key $K_{g}$ cannot compute either $K_{i}$ or $K_{j}$. Even if the adversary eavesdrops past messages, the hash function’s collision resistance prevents the derivation of the inputs from the outputs. Moreover, $V_{2}$ and $V_{4}$ cannot be correctly regenerated without the corresponding ephemeral random values ($r_{u}$, $a_{i}$, $r_{s}$, $b_{j}$). Thus, forging any gateway message is computationally infeasible, and the scheme resists *GW* impersonation.

#### 4.3.3. Sensor Node Impersonation Attack

To impersonate a sensor node $S_{j}$, the adversary must forge the response $\left\{V_{4},m_{3},m_{4},{TS}_{3}\right\}$ to GW. This requires

- The sensor’s NTRU private key $\left(f_{s},f_{s,p}^{-1}\right)$ to decrypt $V_{2}$ and extract $a_{i},U_{IDi},h_{u}$,
- The secret $K_{j}=H\left(SID_{j}∥K_{g}\right)$ to compute $m_{3}=H\left(K_{j}∥SID_{j}∥U_{IDi}∥TS_{3}\right)$,
- The ephemeral nonce $b_{j}$ and blinding factor $r_{s}$ to construct $V_{4}=h_{u}\cdot r_{s}+b_{j}$ and $m_{4}=H\left(b_{j}∥SID_{j}∥U_{IDi}∥{TS}_{3}\right)$.

Without $K_{j}$, the adversary cannot forge $m_{3}$. Without the private key, they cannot recover $a_{i}$ (needed to link the response to the ongoing session). Even if a sensor has been previously captured, the captured private key cannot be used to impersonate a different sensor or to forge messages for a session that originated from a legitimate user who has not yet interacted with the captured node. The scheme therefore resists sensor node impersonation.

#### 4.3.4. Forward Secrecy

In the proposed scheme, the session key is computed as ${SK}_{u-s}\;=\;H\left(a_{i}\left|\right|b_{j}\left|\right|{\mathrm{UID}}_{i}\left|\right|{\mathrm{SID}}_{j}\right)$, where $a_{i},\;b_{j}$ denote ephemeral one-time random nonces separately generated by the user $U_{i}$ and sensor ${\mathrm{SN}}_{j}$ in each independent authentication round. The gateway’s long-term master secret $K_{g}$ only participates in computing static pre-registration secrets $K_{i}\;=\;H\left({\mathrm{RID}}_{i}\left|\right|K_{g}\right)$ and $K_{j}\;=\;H\left({\mathrm{SID}}_{j}\left|\right|K_{g}\right)$ during offline registration, and it is never embedded into the hash derivation formula of the session key. If an adversary fully leaks the gateway’s master key $K_{g}$, he can only deduce the permanent static credentials $K_{i}$ and $K_{j}$ stored in the gateway database. However, the ephemeral values $a_{i}$ and $b_{j}$ are freshly sampled per session and encapsulated inside NTRU ciphertexts $V_{2}$ and $V_{4}$ transmitted over public channels. Breaking NTRU polynomial encryption requires solving the hard ring lattice problem, which is computationally infeasible for classical and quantum polynomial-time adversaries. Without recovering $a_{i}$ and $b_{j}$, the adversary cannot reconstruct the historical session key ${SK}_{u-s}$. Therefore, our scheme provides perfect forward secrecy.

#### 4.3.5. Replay Attack

Every authentication message carries a timestamp: ${TS}_{1}$ in the login request, ${TS}_{2}$ from GW to $S_{j}$, ${TS}_{3}$ from $S_{j}$ to GW, and ${TS}_{4}$ from GW to $U_{i}$. Upon receipt, each party verifies that $\left|{TS}^{*}-TS\right|\leq \Delta T$, where ${TS}^{*}$ is the current time. Any replayed message with a stale timestamp is immediately rejected. Moreover, even if an adversary replays a message within the valid time window, the inclusion of fresh nonces ($a_{i},b_{j},r_{u},r_{s}$) in the hash computations (e.g., $m_{1},m_{4}$) and in the NTRU ciphertexts makes the replayed message inconsistent with the current session’s expected values. The receiving entity will detect the mismatch when verifying the authentication tags. Consequently, replay attacks are effectively prevented.

It should be noted that practical WSN nodes suffer inherent clock drift and clock offset. The configurable threshold *ΔT* can tolerate moderate clock offsets, but overly enlarged *ΔT* will widen the valid time window and increase the replay-attack surface. Hence, system operators need to balance clock-desynchronization robustness and replay-attack resistance in real deployment.

#### 4.3.6. Privileged Insider Attack

A privileged insider (e.g., a gateway administrator) may have full read access to the gateway’s database, which stores $\left\{U_{IDi},K_{i},h_{u}\right\}$ for each user and $\left\{SID_{j},K_{j},h_{s}\right\}$ for each sensor. However, the user’s password $U_{PWi}$ is never transmitted to or stored by the gateway; during registration, the user sends only $U_{IDi}$, $h_{u}$, and $RID_{i}=H\left(U_{IDi}∥x\right)$, where x is a nonce chosen locally by the user. The smartcard stores $B_{i}=H\left(U_{IDi}∥U_{PWi}\right)⊕x$, $C_{i}=K_{i}⊕F_{i}$, and $F_{i}=H\left(U_{PWi}∥x\right)\;mod\;n_{0}$. Even if the insider obtains $K_{i}$ from the database, they cannot recover x or $U_{PWi}$ because both are protected by the one-way hash H. The fuzzy verifier $F_{i}$ further obstructs offline guessing: any guessed $\left(U_{IDi}^{*},U_{PWi}^{*}\right)$ produces a false match with probability $1/n_{0}$, forcing the insider to validate guesses through online interactions, which are limited. Thus, the scheme resists privileged insider attacks.

#### 4.3.7. Sensor Node Capture Attack

Sensor nodes are deployed in unattended and potentially hostile environments, making physical capture a realistic threat. Suppose an adversary captures sensor $S_{j}$ and extracts its stored secrets, including $K_{j}=H\left(SID_{j}∥K_{g}\right)$ and the NTRU private key $\left(f_{s},f_{s,p}^{-1}\right)$ (if stored). The adversary can decrypt past $V_{2}$ messages for that specific node. However,

(1) The session key $SK_{u-s}=H\left(a_{i}∥b_{j}∥U_{IDi}∥SID_{j}\right)$ depends on the user’s ephemeral nonce $a_{i}$, which was encrypted under $h_{s}$. Although the captured private key allows decryption of $V_{2}$, the adversary still obtains $a_{i}$ only for sessions involving the captured node. For other sessions with other sensors, $a_{i}$ is encrypted under different public keys and remains secure.
(2) The gateway’s master key $K_{g}$ is not stored on the sensor; so, capturing one sensor does not reveal $K_{j}$ for other sensors (since $K_{j}=H\left(SID_{j}∥K_{g}\right)$ and $K_{g}$ is unknown).
(3) The ephemeral blinding factor $r_{u}$ is discarded after the session; without it, the adversary cannot forge new valid ciphertexts for future sessions.

Consequently, the capture of a single sensor node does not compromise the security of other nodes or past session keys for non-captured nodes. The scheme resists sensor node capture attacks.

#### 4.3.8. User Anonymity

User anonymity comprises two aspects: (a) the adversary cannot learn the real identity $U_{IDi}$, and (b) the adversary cannot determine whether two different protocol runs originate from the same user (untraceability). In our scheme, $U_{IDi}$ is never sent in cleartext over the public channel:

- In the login request, $V_{1}=H\left(s_{ug}\right)⊕\left(U_{IDi}∥SID_{j}∥{TS}_{1}\right)$ masks the identity with the secret $s_{ug}$, which is known only to the user and the gateway. Without $s_{ug}$, extraction of $U_{IDi}$ is impossible due to the one-wayness of H(). In addition, because the timestamp *TS*_1_ is embedded in *V*_1_, the *V*_1_ for each user login to GWN is different, making it impossible for attackers to track users based on the intercepted *V*_1_.
- The same identity also appears inside the NTRU ciphertext $V_{2}$ as part of the plaintext $\left(a_{i}∥U_{IDi}∥h_{u}\right)$, which is encrypted under the sensor’s public key $h_{s}$. Only the sensor (with its private key) can recover it.
- The nonce $a_{i}$ changes uniformly at random for each session; so, the ciphertext $V_{2}$ and the masked value $V_{1}$ are computationally indistinguishable from random values across different sessions. An adversary cannot link two login requests to the same user. Thus, the protocol guarantees both user anonymity and untraceability.

#### 4.3.9. Quantum Attack

Our protocol’s security foundation rests on the NTRU public-key cryptosystem, which is a lattice-based primitive and the security relies on NTRU’s hardness, which is derived from SVP and CVP in ideal lattices. Moreover, the hash function H() used throughout the protocol (e.g., SHA-256) is also quantum-resistant. The authentication tags $m_{1},m_{2},\ldots ,m_{5}$ are constructed from H() with secret inputs ($K_{i},K_{j},b_{j}$); An adversary would need to invert these hashes or forge them without the secrets, which reduces to breaking the one-wayness or collision resistance of H().

Importantly, the ephemeral nonces $a_{i},b_{j}$ and blinding factors $r_{u},r_{s}$ are chosen independently of any long-term quantum-vulnerable parameter. Therefore, an adversary equipped with a quantum computer cannot trivially recover past session keys (forward secrecy holds) nor impersonate any party without solving the underlying lattice problem. In summary, the proposed scheme is explicitly designed to be post-quantum secure, offering a distinct advantage over ECC-based and Chebyshev chaotic maps-based counterparts.

## 5. Performance Analysis

This section evaluates the performance of our proposed NTRU-based two-factor authentication scheme in terms of computational overhead, storage overhead, and communication overhead. A comprehensive comparison with the four state-of-art schemes [16,17,23,25] is presented to demonstrate the efficiency advantages of our protocol.

### 5.1. Computation Overhead

We analyze the computation cost of our scheme and the related schemes [16,17,23,25] during the authentication phase, focusing on the cryptographic operations performed by the user, the gateway, and the sensor node. Following the benchmark established in [25], we adopt the execution times shown in Table 4. Our scheme mainly involves operations such as NTRU encryption, decryption, hash function, and polynomial multiplication. Operations such as XOR and concatenation are omitted due to their negligible overhead. The comparison results of the computation overhead between our scheme and other schemes [16,17,23,25] are shown in Table 5. To facilitate understanding, we have represented Table 5 graphically in Figure 5.

As shown in Table 5 and Figure 5, the proposed scheme demonstrates superior computational efficiency, achieving the lowest total overhead of 0.4410 ms. It significantly outperforms the ECC-based schemes [16,17] and the chaotic map-based scheme [23]. Compared to the other NTRU-based protocol [25], our design optimizes the gateway’s role by eliminating costly NTRU operations, reducing its workload to only five hash functions. This minimal computational demand makes the protocol exceptionally suitable for resource-constrained WSNs.

Note that the above measured computation overhead is based on our moderate NTRU parameter n. As analyzed in Section 2.2, a larger n for high-security scenarios increases sensor-side cryptographic latency, whereas a smaller n for delay-sensitive IoT applications degrades security margins. Practitioners should tune NTRU parameters following the concrete application security-latency demands.

### 5.2. Storage Overhead

We analyze the storage overhead incurred by each entity during the authentication phase. Following the benchmark established in Section 5.1 of [25], we assume the following bit lengths: identity (64 bits), timestamp (32 bits), nonce (128 bits), hash value (160 bits), NTRU public/private key (128 bits), ECC point (320 bits), symmetric key (128 bits), and biometric data (160 bits). The storage overhead of our solution at the user end is 480 bits (=160 + 160 + 160), at the gateway end it is 704 bits (=64 + 160 + 128 + 64 + 160 + 128), and at the sensor end it is 352 bits (=64 + 160 + 128). The total storage cost comparison results of our scheme with those of schemes [16,17,23,25] are shown in Table 6 and Figure 6.

According to Table 6 and Figure 6, the proposed scheme requires a total storage of 1536 bits, which is the second most efficient among the compared protocols, surpassed only by scheme [17] (1508 bits). It significantly reduces the storage burden compared to schemes [16,23,25]. This analysis highlights that our design achieves a highly competitive storage footprint, effectively balancing the memory requirements across the user, gateway, and sensor nodes for practical implementation on resource-constrained devices.

### 5.3. Communication Overhead

Based on the benchmark proposed in Section 5.2, we calculated the communication cost of our scheme and compared it with other schemes [16,17,23,25]. The comparison results are shown in Table 7 and Figure 7.

Table 7 and Figure 7 show that the communication overhead of the proposed scheme is 1600 bits. While this is slightly higher than schemes [25] (1440 bits) and [23] (1472 bits), it remains substantially more efficient than the ECC-based protocols [16] and [17]. This moderate increase in communication cost is a justifiable trade-off for achieving post-quantum security through the NTRU cryptosystem. The protocol maintains a reasonable level of transmission efficiency while providing robust security against future quantum computing threats.

### 5.4. Summary

In conclusion, the proposed NTRU-based authentication protocol offers a compelling balance between security and performance. It achieves the lowest computational cost and the most efficient storage overhead among the compared schemes, making it highly suitable for resource-constrained WSNs. Although the communication cost is marginally higher than some existing schemes, this is a reasonable trade-off for achieving post-quantum security. The significant advantages in computation and storage, combined with its robust security foundation, establish the proposed protocol as a highly efficient and future-proof solution for WSNs.

## 6. Conclusions

In this paper, we propose a novel user authentication protocol for WSNs based on the NTRU lattice cryptosystem. Addressing the security vulnerabilities of traditional schemes and the impending threats posed by quantum computing, our design ensures robust post-quantum security while maintaining high efficiency. We conduct formal security proof under the random oracle model and also verify the security of our scheme through BAN logic. Informal security analysis indicates that the protocol can effectively resist various known attacks. Furthermore, performance evaluations confirm that our scheme achieves the lowest computational cost and superior storage overhead compared to existing alternatives based on ECC, Chebyshev chaotic map, and NTRU. Despite slightly higher communication costs, the significant improvements in computational efficiency and quantum resistance make the proposed protocol a highly practical and secure solution in resource-constrained WSNs environments.

## Figures and Tables

**Figure 1:**
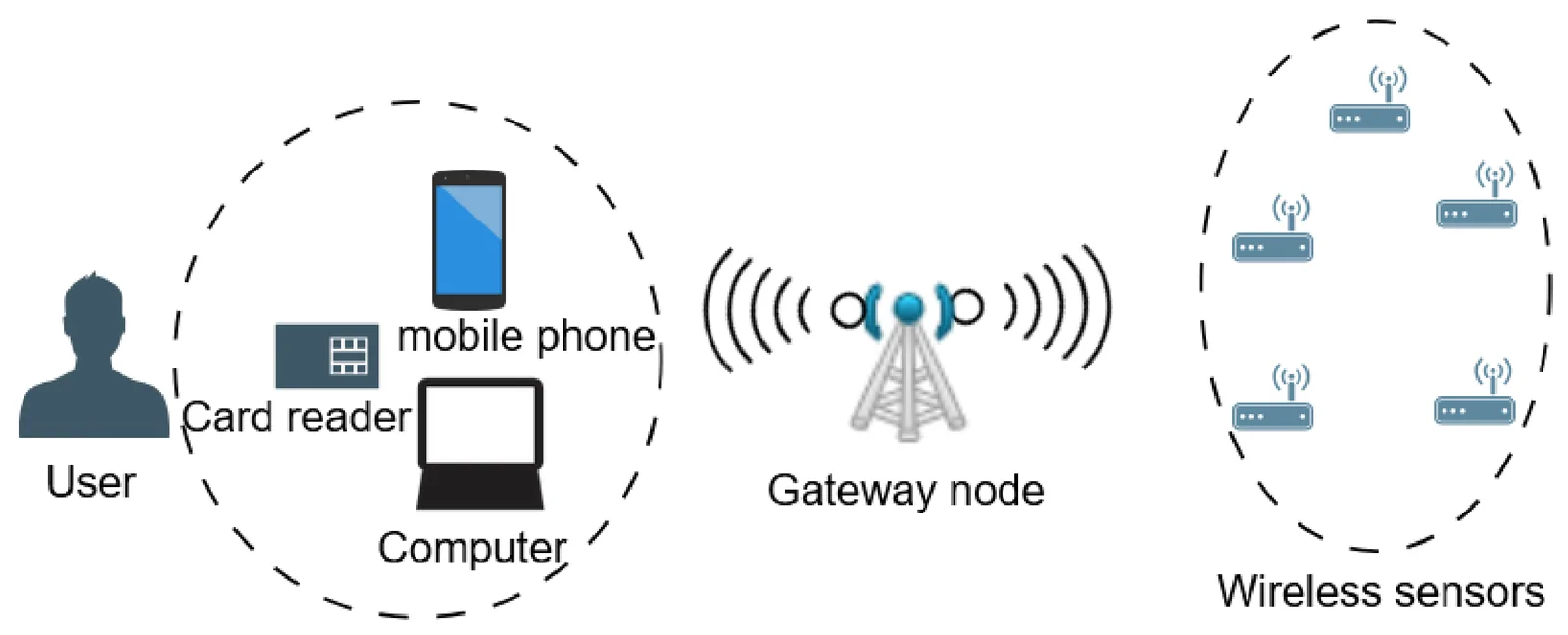
The architecture of WSNs.

**Figure 2:**
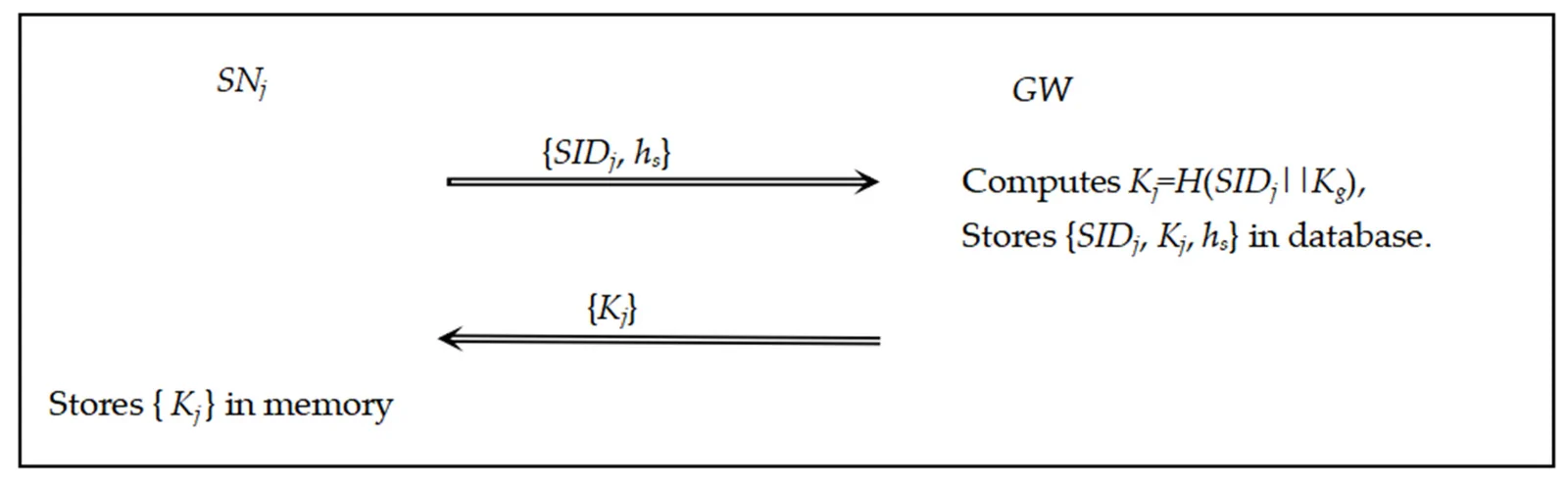
The sensor registration phase.

**Figure 3:**
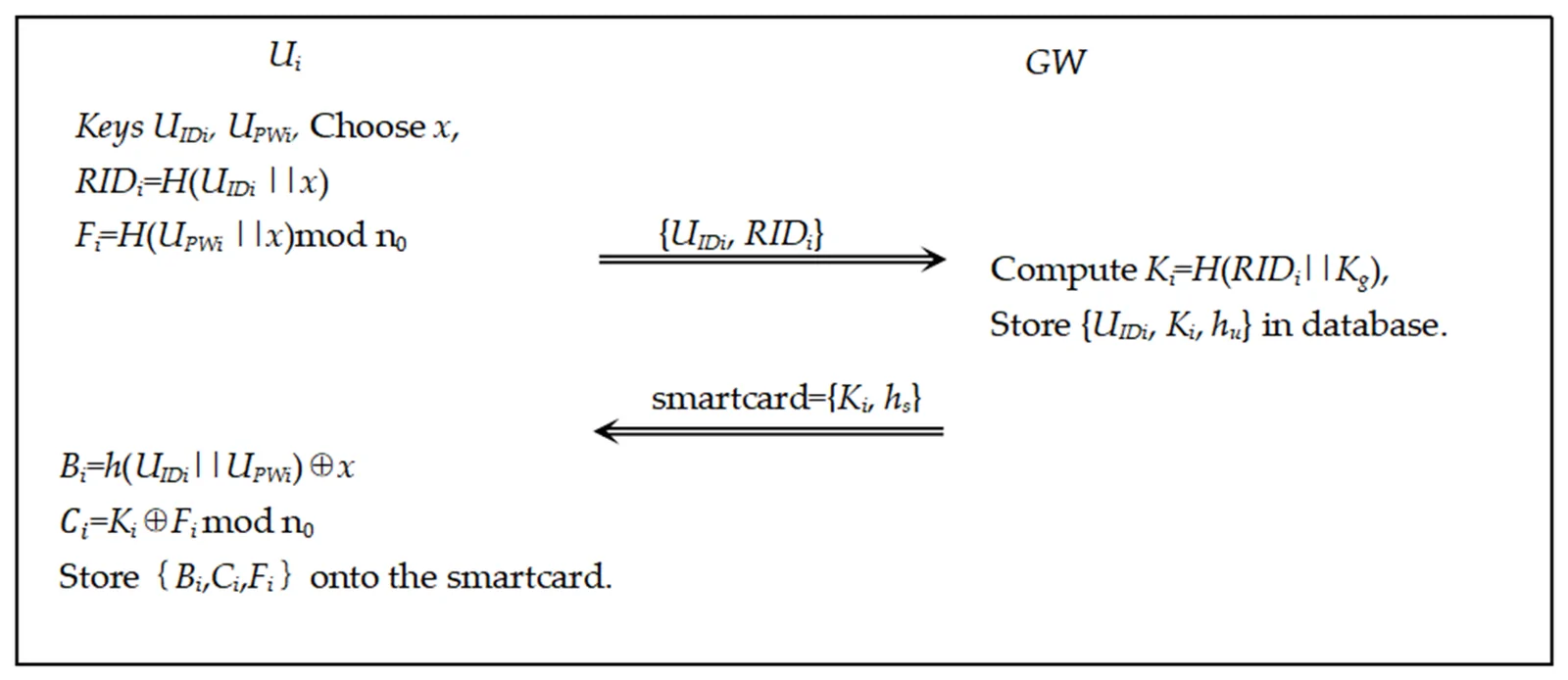
The user registration phase.

**Figure 4:**
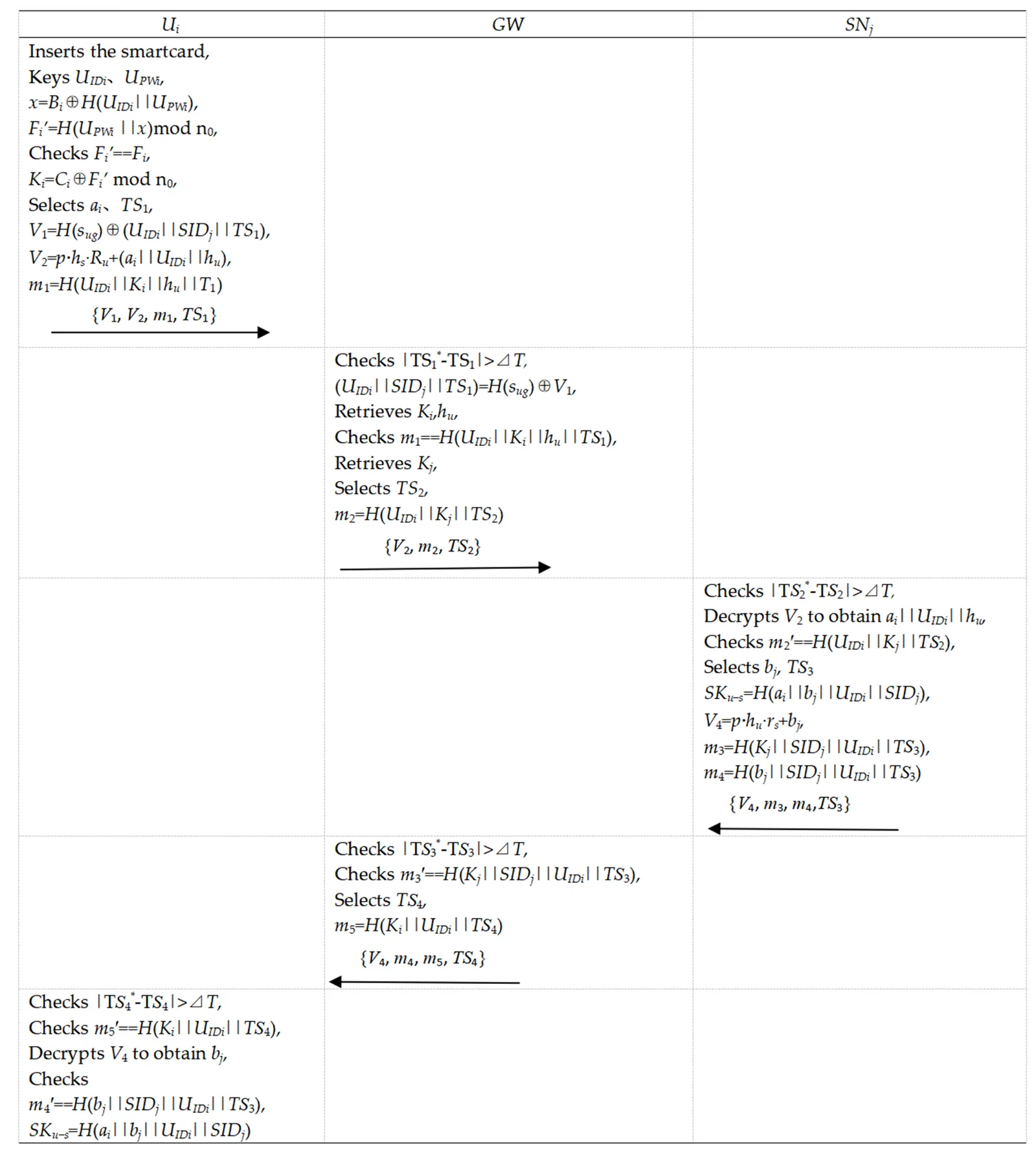
The authentication phase.

**Figure 5:**
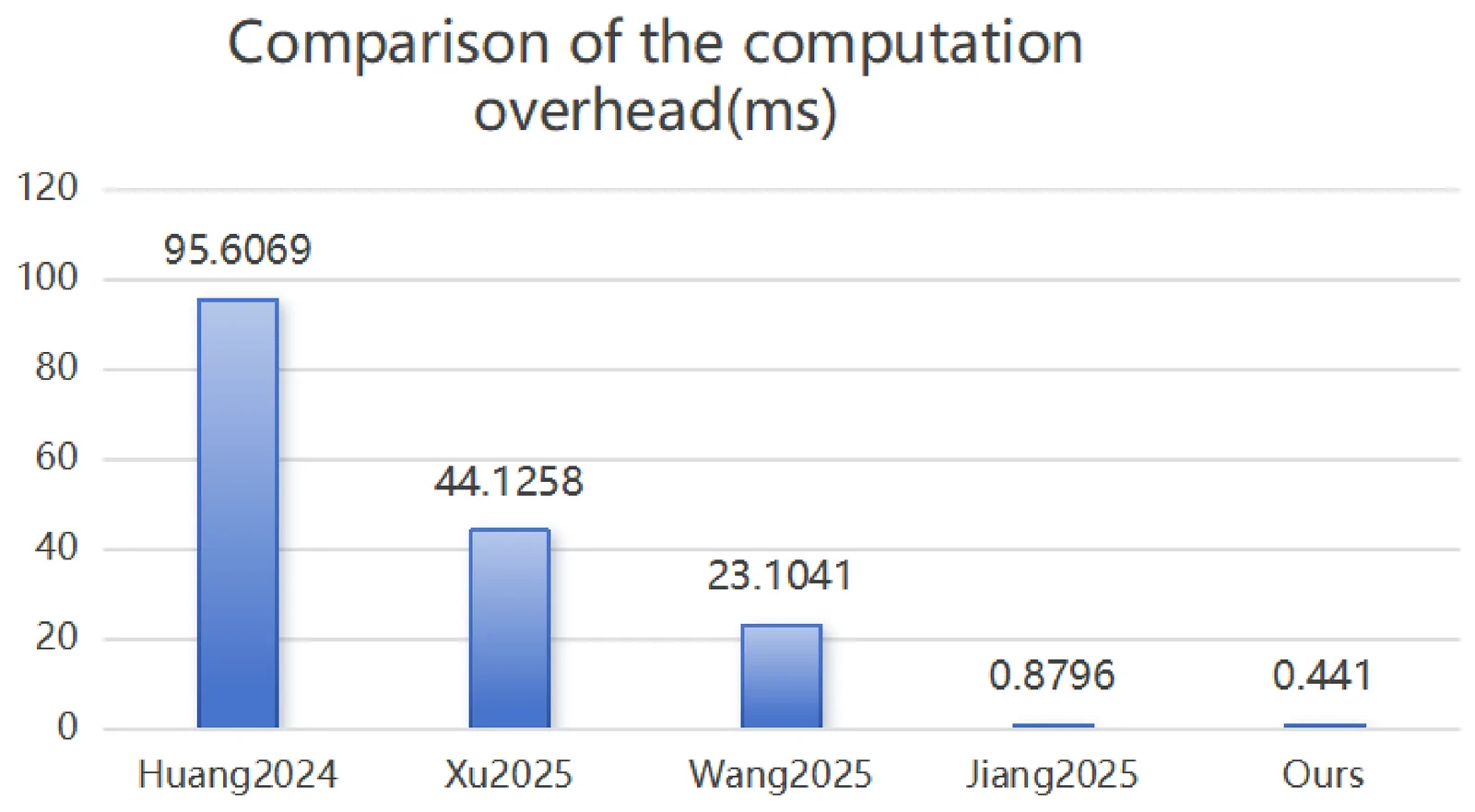
Comparison of the computation overhead [16,17,23,25].

**Figure 6:**
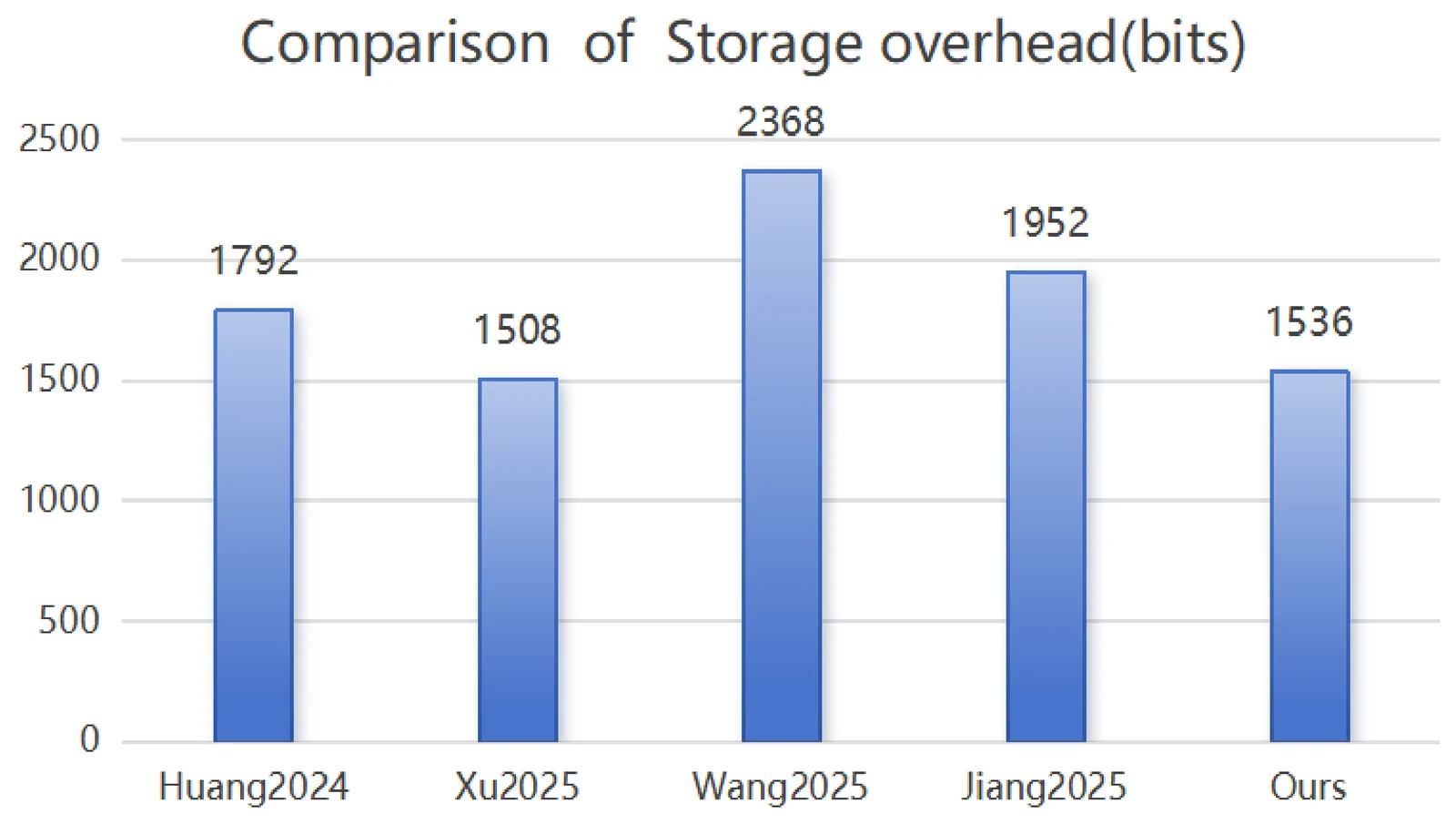
Comparison of storage overhead [16,17,23,25].

**Figure 7:**
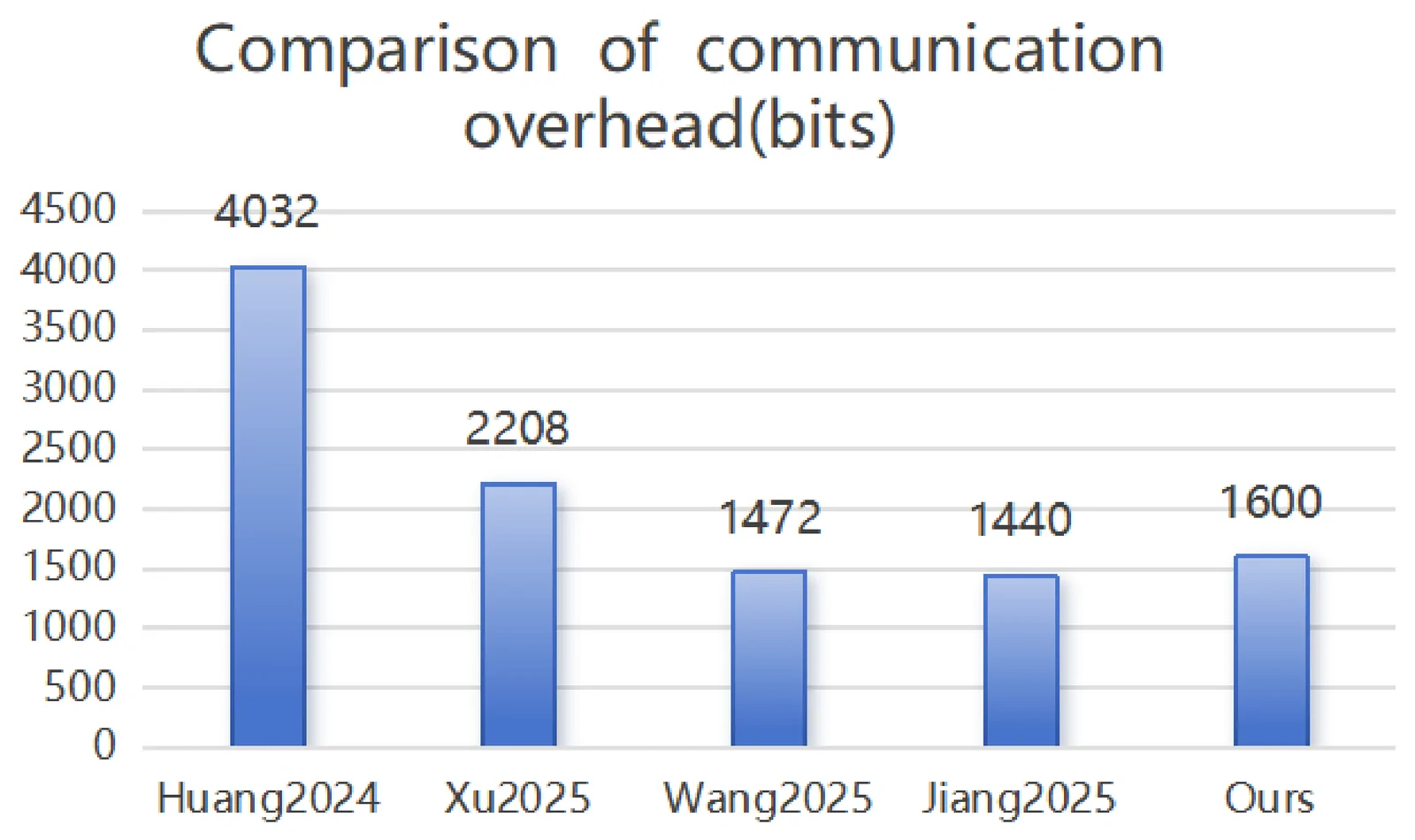
Comparison of communication overhead [16,17,23,25].

**Table 1:** Notation and its meaning.

Notation	Meaning
*U_i_*	The *i*th user
*GW*	The gateway node
*SN_j_*	The *j*th sensor node
*U_IDi_*	Identity of user
*U_PWi_*	Password of user *U_i_*
*SID_j_*	Identity of sensor *SN_j_*
*K_g_*	Master secret key of the gateway
*S_ug_*	Shared secret between *U_i_* and *GW* established during registration
*TS_i_*	Timestamps used in the authentication phase, i = 1,2,3,4
*ΔT*	Maximum allowable transmission delay
*H*()	Secure one-way hash function
⊕	Bitwise XOR operation
||	Concatenation operation
→	Transmission over a public channel
⇒	Transmission over a secure channel

**Table 2:** The practical prerequisites for A1–A21 and their validity in real-world WSN.

Assumption	Practical Prerequisite	Valid in Real-World WSN?
A1	$K_{i}=H\left(RID_{i}∥K_{g}\right)$ is established during user registration over a secure channel; only $U_{i}$ and GW know it; smartcard is tamper-resistant.	Valid under secure registration and tamper-resistant smartcard.
A2	$K_{j}=H\left(SID_{j}∥K_{g}\right)$ is distributed during sensor registration over a secure channel; only $SN_{j}$ and GW know it.	Valid under secure pre-deployment/registration.
A3	$U_{i}$ can correctly recover $K_{i}$ from the smartcard.	Valid if smartcard is secure and password-protected.
A4	$SN_{j}$ securely stores $K_{j}$.	Valid if sensor is tamper-resistant.
A5	$U_{i}$ trusts GW’s jurisdiction over the session key.	Valid if GW is trusted.
A6	$SN_{j}$ trusts GW’s jurisdiction over the session key.	Valid if GW is trusted.
A7	$U_{i}$ trusts $SN_{j}$’s jurisdiction over $b_{j}$; $SN_{j}$’s random source is reliable.	Valid if $SN_{j}$ is tamper-resistant, and its RNG is secure.
A8	$SN_{j}$ trusts $U_{i}$’s jurisdiction over $a_{i}$, and trusts GW’s forwarding of $U_{i}$’s authentication result.	Valid if user device is trusted, and GW is trusted.
A9	GW can verify freshness of ${TS}_{1}$; $U_{i}$ and GW are time-synchronized.	Valid under lightweight time synchronization.
A10	$SN_{j}$ can verify freshness of ${TS}_{2}$; $SN_{j}$ and GW are time-synchronized.	Valid under lightweight time synchronization.
A11	GW can verify freshness of ${TS}_{3}$; GW and $SN_{j}$ are time-synchronized.	Valid under lightweight time synchronization.
A12	$U_{i}$ can verify freshness of ${TS}_{4}$; $U_{i}$ and GW are time-synchronized.	Valid under lightweight time synchronization.
A13	$a_{i}$ is bound with ${TS}_{1}$; GW derives freshness of $a_{i}$ from freshness of ${TS}_{1}$.	Valid when $m_{1}$ covers $a_{i}$.
A14	$b_{j}$ is bound with ${TS}_{4}/m_{4}$; $U_{i}$ derives freshness of $b_{j}$ from freshness of ${TS}_{4}$.	Valid when $b_{j}$ is fresh in each session.
A15	$SN_{j}$ believes $h_{u}$ is $U_{i}$’s public key, and the corresponding private key is known only to $U_{i}$.	Valid if public keys are pre-loaded or authenticated.
A16	$U_{i}$ believes $h_{s}$ is $SN_{j}$’s public key.	Valid if public keys are pre-loaded or authenticated.
A17	$SN_{j}$ securely stores its NTRU private key $\left(f_{s},f_{s}^{i}\right)$.	Valid if sensor is tamper-resistant.
A18	$U_{i}$ securely stores its private key $\left(f_{u},f_{u}^{i}\right)$.	Valid if smartcard is tamper-resistant.
A19	GW securely stores $\left\{U_{IDi},K_{i},h_{u}\right\}$, with correct binding between identity and public key.	Valid under secure registration.
A20	GW securely stores $\left\{SID_{j},K_{j},h_{s}\right\}$, with correct binding between identity and public key.	Valid under secure registration.
A21	$s_{ug}$ is a long-term shared secret established during registration and shared between $U_{i}$ and GW; its freshness is provided by ${TS}_{1}$, not by $s_{ug}$ itself.	Valid under secure registration. The original $\#\left(s_{ug}\right)$ assumption is unreasonable and has been corrected.

**Table 3:** Security features comparison among schemes.

Security Features	[16]	[17]	[23]	[25]	Ours
User impersonation resistance	✓	✓	✗	✗	✓
GW impersonation resistance	✓	✓	✓	✓	✓
Sensor node impersonation resistance	✓	✓	✓	✓	✓
Forward secrecy	✓	✓	✓	✓	✓
Replay attack resistance	✓	✗	✓	✓	✓
Privileged insider attack resistance	✓	✓	✓	✓	✓
Sensor node capture attack resistance	✗	✓	✓	✓	✓
User anonymity	✓	✓	✓	✓	✓
Quantum attack resistance	✗	✗	✗	✓	✓

**Table 4:** Cryptographic operations and their execution times.

Symbols	Meanings	Time (ms)
*T_h_*	Hash function	0.0004
*T_c_*	Chebyshev polynomial	2.45
*T_M_*	ECC point multiplication	7.3529
*T_E_*	ECC encryption/decryption	7.3529
*T_FE_*	Fuzzy extractor	7.3529
*T_se_*	Symmetric encryption/decryption	0.1303
*T_Ne_*	NTRU encryption	0.13
*T_Nd_*	NTRU decryption	0.086
*T_mm_*	Polynomial multiplication	0.0011

**Table 5:** The comparison results of the computation overhead.

Scheme	*U_i_*	*GW*	*SN_j_*	Total (ms)
Huang 2024 [16]	5*T_M_* + 22*T_h_* + *T_FE_*	4*T_M_* + 18*T_h_*	3*T_M_* + 8*T_h_*	95.6069
Xu 2025 [17]	3*T_M_* + 9*T_h_*	*T_M_* + 7*T_h_*	2*T_M_* + 5*T_h_*	44.1258
Wang 2025 [23]	8*T_h_* + 2*T_se_* + 2*T_c_* + *T_FE_*	10*T_h_* + 4*T_se_* + 3*T_c_*	4*T_h_* + 2*T_se_* + *T_c_*	23.1041
Jiang 2025 [25]	9*T_h_* + *T_Ne_* + *T_Nd_* + *T_mm_*	13*T_h_* + 2*T_Ne_* + 2*T_Nd_* + 2*T_mm_*	6*T_h_* + *T_Ne_* + *T_Nd_* + *T_mm_*	0.8796
Ours	8*T_h_* + *T_Ne_* + *T_Nd_* + *T_mm_*	5*T_h_*	4*T_h_* + *T_Ne_* + *T_Nd_* + *T_mm_*	0.4410

**Table 6:** Storage overhead.

Scheme	*U_i_*	*GW*	*SN_j_*	Total (Bits)
Huang 2024 [16]	1120	448	224	1792
Xu 2025 [17]	928	320	160	1508
Wang 2025 [23]	768	1120	480	2368
Jiang 2025 [25]	928	544	480	1952
Ours	480	704	352	1536

**Table 7:** Communication overhead.

Scheme	Huang2024	Xu2025	Wang2025	Jiang2025	Ours
Communication overhead(bits)	4032	2208	1472	1440	1600

## Data Availability

All data generated or analyzed through the paper are associated with their references and sources.
